# High-sensitivity plasma proteomics reveals disease-specific signatures and predictive biomarkers of Alzheimer’s disease phenotypes in a large mixed-dementia cohort

**DOI:** 10.1186/s13024-025-00909-x

**Published:** 2025-11-17

**Authors:** Katherine Gong, Jigyasha Timsina, Muhammad Ali, Yike Chen, Menghan Liu, Ciyang Wang, Cyril Pottier, Geoffrey K. Feld, Gyujin Heo, Tammie L. S. Benzinger, Cyrus A. Raji, Beau Ances, Brian A. Gordon, Julie K. Wisch, Suzanne E. Schindler, John C. Morris, David M. Holtzman, Laura Ibanez, Carlos Cruchaga

**Affiliations:** 1 Department of Psychiatry, Washington University School of Medicine, 4444 Forest Park Ave, St. Louis, MO 63110 USA; 2 Neurogenomics and Informatics Center, Washington University School of Medicine, St. Louis, MO 63110 USA; 3 Division of Biology and Biomedical Sciences, Washington University School of Medicine, St. Louis, MO USA; 4 Department of Neurology, Washington University School of Medicine, St. Louis, MO 63110 USA; 5 Geocyte LLC, Dublin, OH USA; 6 Department of Genetics, Washington University School of Medicine, St. Louis, MO 63110 USA; 7 Department of Radiology, Washington University School of Medicine, St. Louis, MO 63110 USA; 8 Knight Alzheimer Disease Research Center, Washington University School of Medicine, St. Louis, MO 63110 USA; 9 Hope Center for Neurologic Diseases, Washington University St. Louis, St. Louis, MO 63110 USA

**Keywords:** Plasma assays, Neurodegeneration diagnostics, Plasma-based diagnostics, CNS protein profiling

## Abstract

Novel plasma assays enabled accurate blood-based biomarkers for neurodegenerative diseases with minimally invasive options for clinical use. Large-scale studies encompassing multiple neurodegenerative diseases using novel multiplex platforms are essential to uncover disease-specific biomarkers and pathways. We generated and analyzed plasma biomarker data using the NULISAseq™ CNS Panel from 3,232 participants with Alzheimer disease (AD), Dementia with Lewy bodies (DLB), Frontotemporal dementia (FTD), Parkinson disease (PD) and cognitively unimpaired individuals, from the Charles F. and Joanne Knight Alzheimer Disease Research Center. We identified proteins associated with disease status and AD-related phenotypes (Clinical Dementia Rating®, CSF Aβ42/Aβ40, Amyloid-PET, and Tau-PET). These proteins were used to identify disease-specific biomarkers and perform pathway analyses. We identified 81 proteins associated with AD, 21 with DLB, four with FTD, and 52 with PD after multiple test correction. Disease comparison showed that PD and DLB had the highest similarity, followed by AD and DLB. Concurrently, each disease also presented disease-specific signatures. Some AD-specific proteins included p-tau217; MSLN and SAA1 were specific to DLB, and FLT1 and PARK7 to PD. We also identified eight proteins associated with Amyloid-PET, eight with Tau-PET, 14 with CSF Aβ42/40 ratio, and 72 with CDR, some of which were specific to each phenotype. We used a data-driven approach to identify the p-tau217 cut-off for biomarker positivity. Plasma p-tau217 achieved an AUC of 0.81 (95% CI: 0.79–0.83) for AD diagnosis and 0.96 (95% CI: 0.94–0.98) for Amyloid positivity. P-tau217 had 93.77% agreement with Amyloid-PET status. Proteins associated with AD were enriched in protein-lipid complex binding pathway, whereas PD associated proteins were enriched in laminin-related pathways. FTD associated proteins were enriched in cytoskeleton proteins. This is the largest plasma NULISA CNS study performed till date and covers the four major neurodegenerative diseases: AD, PD, DLB and FTD. We validated the high classification accuracy of the NULISA plasma p-tau217 and its strong correlation with Amyloid-PET status. We also identified disease-specific proteins that could enhance differential diagnosis. These findings highlight the potential of the NULISA platform as a reliable quantitative tool for research and clinical applications in neurodegenerative diseases.

**Supplementary Information:**

The online version contains supplementary material available at 10.1186/s13024-025-00909-x.

## Introduction

Neurodegenerative diseases such as Alzheimer disease (AD), Parkinson disease (PD), frontotemporal dementia (FTD), and dementia with Lewy bodies (DLB) affect millions of people worldwide. With the increase in global life expectancy, the socio-economic burden of neurological disorders is rising [1–3]. Although many types of therapeutic targets have been evaluated [4], the only disease-modifying treatment that have demonstrated definitive efficacy in slowing cognitive decline thus far are anti-amyloid antibodies that target amyloid pathology [5]. To enable development of additional types of treatments, novel biomarkers are needed to identify individuals with different pathologies. Biomarkers requiring Positron Emission Tomography (PET) scans or cerebrospinal fluid (CSF) are burdensome to patients and providers and require specialized personnel and/or equipment. Blood-based biomarkers are practically more useful because of their minimal invasiveness and the potential for integration into routine clinical practice [6, 7].

Numerous studies have explored the use of plasma biomarkers in neurodegenerative diseases [8–13]. Plasma p-tau217 has demonstrated high accuracy in detecting AD pathophysiology within the context of routine clinical practice in a memory clinic [8]. In addition, plasma biomarkers such as the ratio of amyloid beta (Aβ42/Aβ40), neurofilament light (NEFL/NfL), and glial fibrillary acidic protein (GFAP) hold significant potential for AD diagnosis and monitoring [9, 10]. Similarly, plasma proteomic analysis in PD has revealed novel biomarkers, such as DOPA decarboxylase (DDC). However, plasma DDC concentrations may be impacted by some medications [11]. The heterogeneity and low prevalence of FTD has hindered the identification of clinically useful biomarkers. Nevertheless, recent research suggests that plasma extracellular vesicles (EVs) content may serve as potential diagnostic biomarkers [12]. Chatterjee et al. demonstrated that EVs in plasma contain quantifiable amounts of TAR DNA-binding protein (TDP-43) and tau isoforms, with distinct patterns observed in diseases like Amyotrophic lateral sclerosis (ALS) and FTD. Their findings show that EV TDP-43 levels and 3R/4R tau isoform ratios effectively discriminate between diagnostic groups and strongly correlate with disease severity, highlighting their potential as biomarkers for FTD disease monitoring [12]. DLB is a common form of cognitive neurodegenerative disease, yet only one-third of patients receive accurate diagnoses, primarily due to its clinical similarities with AD and PD. Several studies have proposed p-tau181 as a potential plasma biomarker for DLB, while AD non-specific biomarkers such as NEFL and GFAP have also shown increased levels across the disease continuum [13].

However, most biomarker studies have focused on a single disease and examined only a limited set of plasma protein markers. There is a pressing need for large-scale studies that compare multiple plasma biomarkers across AD, PD, DLB, and FTD, while also identifying novel disease-specific biomarkers. The newly developed NUcleic acid Linked Immuno-Sandwich Assay (NULISA) platform offers an optimal balance between multiplexing capability, low sample volume, and accuracy. It has limited cross-reactivity issues, commonly seen in other multiplexed immunoassay platforms [14]. We have previously demonstrated the reliability of the NULISA measurements for biomarker measurement in AD in a small dataset (*n* = 48), showing strong correlations with established assays in both CSF and plasma [15]. Additionally, another research group has reported similar findings (*n* = 176) supporting NULISA's accuracy [16]. In this study, we aimed to leverage the multiplexing capability of this platform to explore proteomic abundance patterns across various neurodegenerative diseases, identify novel dysregulated proteins, and assess their potential as biomarkers. We utilized the NULISA CNS Disease Panel, which includes validated AD biomarkers (p-tau217, p-tau181, TREM2) alongside key proteins associated with PD (SNCA, pSNCA-129), FTD (TDP-43), and general neurodegeneration (NEFL, GFAP, NRGN, SMOC1). Our large, well-characterized cohort allowed us to evaluate the predictive power of these proteins across diseases and identify novel disease-specific biomarkers. In addition, we also identified plasma proteins associated with AD phenotypes such as Amyloid-PET, Tau-PET, clinical dementia rating (CDR) and CSF Aβ42/Aβ40 ratio. Through pathway analysis, we sought to uncover the biological mechanisms driving disease progression. Finally, we assessed the platform's reproducibility and cross-validated its performance against other orthogonal platforms.

## Materials and methods

### Ethics statement

The study was approved by the Institutional Review Board of Washington University School of Medicine in St. Louis (IRB #201109148) and conducted in accordance with approved protocols. Ethics approval for individual cohorts was obtained from their respective IRBs, with written informed consent provided by participants or their families

### Study design

In this study, we investigated alterations in the plasma proteome to identify proteomic signatures associated with four different neurodegenerative diseases (AD, FTD, DLB and PD). We measured 123 unique proteins included in the NULISAseq™ CNS Disease Panel. A total of 4,400 human plasma samples were collected, and after quality control (QC), 3,947 samples from 3,232 unique participants remained. These participants were enrolled at the Charles F. and Joanne Knight Alzheimer Disease Research Center (Knight-ADRC). To identify disease-specific proteomic changes, differential abundance analysis was performed comparing each disease group (*n*_AD_ = 1,123; *n*_DLB_ = 75; *n*_FTD_ = 58; *n*_PD_ = 126) to healthy controls (CO; *n*_CO_ = 1,596). Significant proteins were then used as input to perform pathway analysis. Similar analyses were conducted for four AD-related phenotypes in a cross-sectional comparison between the clinical AD and CO groups: Amyloid-PET (*n* = 313), Tau-PET (*n* = 266), CSF Aβ42/Aβ40 (*n* = 529), and Clinical Dementia Rating (CDR; *n* = 2,719). Finally, we evaluated the inter-experiment reproducibility of results using 86 identical samples and the correlation of protein measurement with other proteomic measurement technologies like immunoassay (*n* = 1,466) and SomaScan (*n* = 3,716).

### Cohort

#### Sample collection

Plasma samples were immediately processed after collection and stored at −80 °C until use. Prior to measurement, samples were thawed and centrifuged at 10,000 g for 10 min. Then, 10 μL supernatant from each sample was plated in 96-well plates and assayed with the NULISAseq^TM^ CNS Disease Panel.

### Proteomics data measurement and quality control

Protein expression levels were measured using the NULISAseq CNS panel, which targets 123 proteins, as previously described [15]. Protein concentrations, reported in NULISA Protein Quantification (NPQ) units, are normalized for intraplate and intensity variability, then log2-transformed to approximate a normal distribution.

As part of the QC procedure, data points outside 1.5 times the interquartile range (IQR) from the first (Q1) or the third (Q3) quartile were flagged as outliers and replaced with NAs. Subsequently, call rates (the proportion of successful measurements) were calculated for each analyte and sample. A two-step call rate threshold filter was then applied, starting with 65% and followed by 85%, to identify and remove relatively low-quality data. The call rate was recalculated after the 65% (high rate of missingness) filter and prior to applying the 85% threshold. This two-step approach was designed to retain borderline analytes and samples. The final dataset consisted of 123 protein analytes and 3,947 samples (3,232 unique participants) after QC. Limit of detection (LOD) and coefficient of variation (CV) were assessed for each analyte; however, these parameters were not used as filters. Following QC, the default log2-based NPQ values were back-transformed to linear NPQ to allow end users to apply their preferred normalization. For this study, protein levels, expressed as linear NPQ values, were log10-transformed and z-score normalized prior to analysis.

### Neuroimaging methods

Magnetic resonance images (MRI) were obtained on 3T Siemens scanners. T1-weighted scans were segmented using FreeSurfer 5.3 (Martinos Center for Biomedical Imaging, Charlestown, Massachusetts, USA) using the Desikan-Killiany atlas. Positron emission tomography (PET) scanning for amyloid was performed using either [11C] Pittsburgh Compound B (PiB) and [18F] Florbetapir, following previously described methods [17], and was processed using the MR-Free pipeline [18]. Individuals were classified as amyloid-positive if they had a cortical amyloid burden above 20 Centiloids (CL) [19]. PET scanning for tau was performed using [18F] Flortaucipir following previously described methods [20]. Images were processed using the publicly available PET unified pipeline (PUP, https://github.com/ysu001/PUP) [17, 21]. Individuals were considered tau positive if they had a partial-volume corrected SUVR greater than 1.5 in the tau signature region (arithmetic mean of tau SUVR in the amygdala, entorhinal cortex, inferior parietal region, and lateral occipital cortex) [22]. Cerebellar grey matter served as the reference region for all PET image processing.

### Cerebrospinal fluid collection

CSF samples were collected at approximately 8:00 AM following overnight fasting. [23, 24] CSF Aβ40 and Aβ42 concentrations were measured by chemiluminescent enzyme immunoassay using a fully automated platform (LUMIPULSE G1200, Fujirebio, Malvern, PA, USA). Individuals were considered amyloid positive if their CSF Aβ42/Aβ40 ratio was less than 0.0673 [23].

## Statistical analysis

### Differential abundance analysis

Proteins associated with each disease were identified using linear regression analysis, with clinical cases (AD, DLB, FTD, and PD) compared to a single clinical control group, which served as the reference group in all comparisons. The regression model was adjusted for age at plasma collection, sex, and the first two surrogate variables (SVs) to account for potential confounding factors. SVs were calculated using the “sva” function from the “sva (v 3.52.0)” package in R, with missing expression values imputed via random sampling with replacement [25]. Differential abundance analysis (DAA) results were used to compare protein effect sizes between each pair of clinical groups via two-tailed z-tests. The z-statistic was calculated as the difference in estimated effects divided by the square root of the summed squared standard errors. P-values were derived from the standard normal distribution and adjusted using the Benjamini-Hochberg (BH) method to control the false discovery rate (FDR) in multiple testing. A protein was considered significant if it had an FDR adjusted p-value < 0.05. Similar linear regression model was also implemented for four AD-related phenotypes: Amyloid-PET, Tau-PET, CSF Aβ42/Aβ40, and CDR. Pearson's correlation was used to compare protein effect sizes across analyses, separately within the disease and phenotype comparison.

For the cross-disease comparison, proteins significant in at least one disease were selected, and their effect sizes were compared. Hierarchical clustering of rows and columns was performed using Euclidean distance and complete linkage. We also implemented a scorecard system to rank the 123 proteins based on their association with all four neurodegenerative diseases. Each protein was assigned a score based on the direction and significance of effect size from the DAA results. A score of + 1 was assigned if the protein showed positive and nominally significant (*p* < 0.05) association with the disease, and +2 if the association was significant after multiple test correction (*FDR-adjusted p* < 0.05). Conversely, a score of −1 or −2 was assigned if the protein had nominally significant or FDR-significant negative association with the disease, respectively. For proteins that did not show a significant association, a score of 0 was assigned. The final score for each protein was calculated as summation of individual scores from each disease group, with a higher score indicating a consistent, significant association across diseases. Because some proteins showed diverging direction of association between diseases, we also calculated an absolute score, where effect size direction was not considered.

### Modeling plasma p-tau217 as a continuous biomarker

Given the non-normal distribution of plasma p-tau217 values, we applied non-parametric approaches to evaluate differences across diagnostic groups. We first used the Kruskal–Wallis test to evaluate overall variation among the five groups (CO, AD, DLB, FTD, and PD). To identify group differences involving AD, we conducted Dunn’s test for multiple comparisons, focusing on pairwise contrasts between AD and each of the other groups. The p-values were adjusted using the Bonferroni correction to control for multiple testing. To assess the discriminative power of plasma p-tau217, we performed logistic regression for five binary outcomes (Amyloid-PET [cutoff = 20] [19], AD, DLB, FTD, PD), adjusting for age and sex. ROC curves were generated from predicted probabilities, and AUCs with 95% confidence intervals were computed using DeLong's method.

### Plasma p-tau217 cutoff determination

To identify robust p-tau217 cutoffs for classifying biomarker positivity, we applied a Gaussian Mixture Model (GMM) clustering approach using the “mclust (v6.0.0)” package [26, 27] in R, a method we have previously shown to be effective in identifying data-driven cutoffs [28]. Briefly, GMM was applied to the z-scored p-tau217 values to identify the two underlying normal distributions within the dataset. The cutoff was set at the z-score value where a sample was equally likely to belong to either distribution.

In addition to the data-driven GMM approach, we also derived a single threshold using Youden’s Index, which balances sensitivity and specificity to optimize classification performance. Previous studies have suggested that a two-cutoff approach with an intermediate group can improve classification accuracy [29, 30]. Accordingly, we derived upper and lower cutoffs for plasma p-tau217 corresponding to 95% sensitivity and specificity, respectively. An Amyloid-PET value of 20 was used to categorize samples as positive ( > 20) or negative ( < 20), as previously reported for Knight ADRC samples [19]. Similarly, we applied this strategy to determine the p-tau217 cutoff for Tau-PET, using a threshold of 1.5 [22] to define Tau PET levels. The concordance of these p-tau217 cutoffs with Amyloid-PET and Tau-PET statuses were then evaluated to ensure robustness.

### Progression to symptomatic AD

We performed a survival analysis using Cox proportional hazards regression model to identify proteins that are associated with progression of individuals to symptomatic AD. The analysis was adjusted for age at plasma draw and sex and was implemented through the “survival (v3.5.5)” R package. Individuals who were cognitive unimpaired at the time of sample collection but progressed to symptomatic AD during follow-up were compared against those who remained unimpaired, with the latter group as the reference. Time-to-event was calculated from the difference between the age at blood draw and the age at last follow-up (for controls) or the age of AD onset (for cases). Significance was defined by an FDR-adjusted p-value < 0.05.

Additionally, samples were classified into high and low groups based on the GMM p-tau217 cutoff (see previous section), and survival times between the groups were compared using the adjusted Curves R package [31, 32]. To determine the discriminative power of p-tau217 between progressors and non-progressors, ROC curves and AUC values [33] were calculated using a multivariate logistic regression adjusted by age and sex, using dichotomized p-tau217 as predictor of clinical status in 5-, 10-, and 15-year intervals.

### Pathway analysis

Pathway enrichment analysis was conducted using the “clusterProfiler (v4.12.0)” package in R [34, 35]. The “enrichGO” function within the package was used to identify overrepresented pathways based on Gene Ontology (GO) annotations. For this analysis, we used the FDR-significant proteins identified in the cross-disease differential abundance analysis. The Entrez gene IDs corresponding to genes matching the protein list were used as input. Significant pathways were defined as those that passed FDR-adjusted *p* < 0.05. Fold enrichment was calculated as the ratio of the proportion of differentially abundant proteins in each pathway to the proportion of all background proteins annotated to that pathway.

### Inter-experiment variability and inter-platform correlation

To assess the technical robustness and reproducibility of the NULISAseq CNS Disease Panel, measurements were first evaluated across multiple runs (*n* = 86) and under varying matrix conditions (*n* = 43; paired samples collected in Ethylenediaminetetraacetic acid (EDTA) and sodium citrate). Further comparisons were performed by correlating NULISAseq measurements with those from SomaScan (*n* = 3,716) and immunoassay (*n* = 1,466). Proteins included in the analysis were based on data availability or presence in both platforms being compared, with common proteins identified based on their UniProt ID. The reproducibility of measurements and comparison of the NULISA platform's performance to other proteomic platforms were assessed using Pearson's correlation coefficient. Significant correlations were defined as those with *p* < 0.05.

## Results

### Study participants

**Fig. 1:**
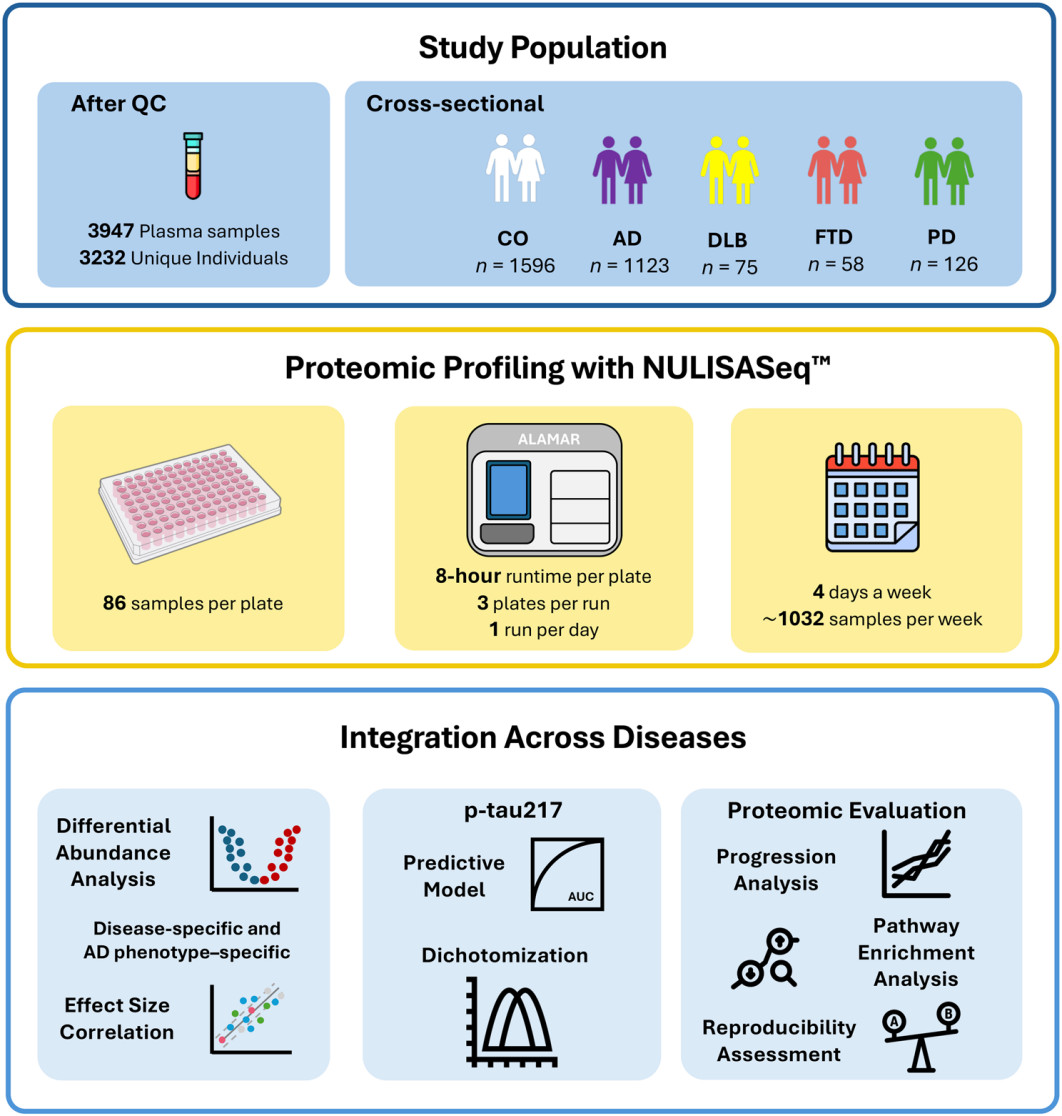
Experimental pipeline for multi-disease proteomic analysis. Overview of the study design utilizing the NULISASeq™ platform, including the study population, key technical features of NULISASeq™, and the statistical analyses performed across multiple disease cohorts

This study included 3,947 plasma samples collected from 3,232 unique participants recruited at the Knight ADRC at Washington University in St. Louis (WashU). These 3,232 participants included 1,123 AD cases, 75 DLB cases, 58 FTD cases, 126 PD cases, and 1,596 cognitively unimpaired controls (CO) based on the diagnosis at the last visit (Fig. 1; Table 1). No batch effect was observed in the proteomic measurements obtained from these samples after QC. The mean age (±standard deviation; *SD*) at plasma draw was 77.39 ± 8.54 for AD, 75.15 ± 8.14 for DLB, 71.66 ± 8.52 for PD, 67.34 ± 9.15 for FTD, and 72.81 ± 10.56 for CO. The proportion of females varied across groups, with the highest percentage observed in the CO group (59.71%), followed by AD (55.83%), PD (36.51%), FTD (36.21%), and DLB (33.33%). The mean age of disease onset (±SD) was 72.83 ± 9.06, 69.21 ± 8.76, 62.31 ± 9.35, and 62.78 ± 9.80 for AD, DLB, PD, and FTD, respectively. The prevalence of *APOE4+* participants was highest in the AD group (58.41%), followed by DLB (50.67%), FTD (37.93%), CO (30.58%), and PD (26.98%; Table 1). Additional AD-related phenotypes included Amyloid-PET (*n* = 313), Tau-PET (*n* = 266), CSF Aβ42/Aβ40 (*n* = 529), and CDR® (*n* = 2,719, Table 2).

**Table 1:** Demographic information of participants in the disease analysis

Group	n	Mean Age at Draw (SD)	Mean Age at Onset (SD)	Female (%)	APOE4+ (%)
AD	1,123	77.39 (±8.54)	72.83 (±9.06)	55.83	58.41
DLB	75	75.15 (±8.14)	69.21 (±8.76)	33.33	50.67
PD	126	71.66 (±8.52)	62.31 (±9.35)	36.51	26.98
FTD	58	67.34 (±9.15)	62.78 (±9.80)	36.21	37.93
CO	1,596	72.81 (±10.56)	-	59.71	30.58

Table 1 summarizes the demographic characteristics of participants in the disease analysis, including the number of participants (n), mean age at the time of sample collection (Age at plasma Draw), mean age at disease onset (Age at Onset), percentage of females in the group (Female %), and percentage of participants carrying at least one *APOE4* allele (*APOE4+* %). Groups include individuals diagnosed with Alzheimer Disease (AD), Dementia with Lewy Bodies (DLB), Parkinson Disease (PD), Frontotemporal Dementia (FTD), and cognitively unimpaired controls (CO)

**Table 2:** Demographic information of participants in the phenotype analysis

Group	n	Mean Age at Draw (SD)	Mean Age at Onset (SD)	Female (%)	APOE4+ (%)
Amyloid-PET	313	68.32 (±8.42)	71.97 (±9.02)	54.31	39.30
Tau-PET	266	68.00 (±8.50)	67.40 (±8.61)	54.14	41.73
CSF Aβ42/Aβ40	529	68.06 (±8.69)	70.30 (±8.26)	56.33	40.64
CDR	2,719	74.70 (±10.03)	73.85 (±9.75)	58.11	42.07

Table 2 presents the demographic characteristics of participants included in the phenotype analysis. The table includes the number of participants (n), mean age at sample collection (Age at Draw), mean age at disease onset (Age at Onset), percentage of females (Female %), and percentage of participants with at least one *APOE4* allele (*APOE4+* %). Phenotypes analyzed include Amyloid-PET values from Amyloid-PET imaging, CSF Aβ42/Aβ40 ratio, Tau-PET imaging results, and Clinical Dementia Rating (CDR)

### Proteomic signatures in plasma across neurodegenerative disease

**Fig. 2:**
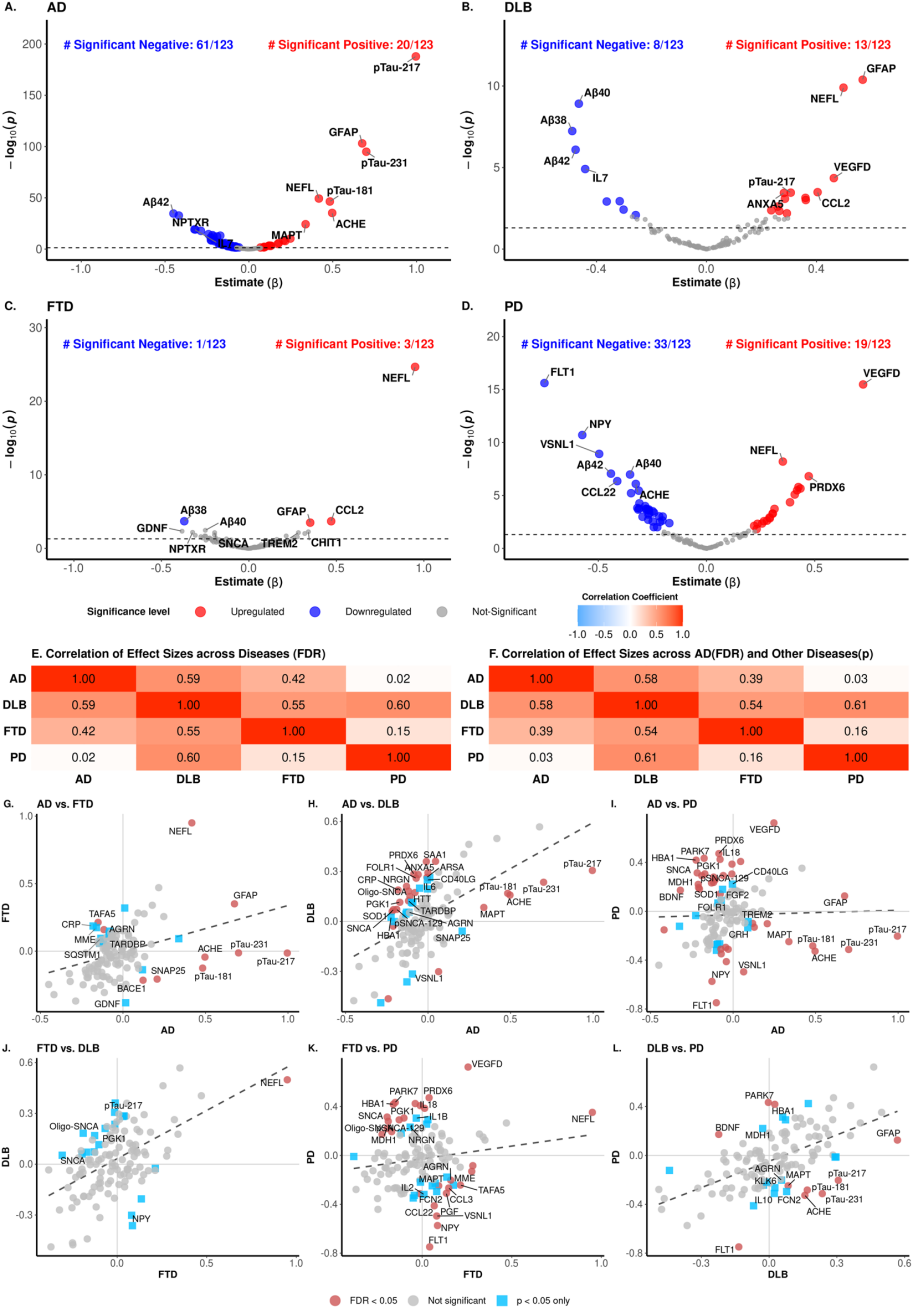
Plasma proteins associated with AD and ADRD. (**A**–**D**) the volcano plots illustrate proteins, selected from a panel of 123, that show significantly different abundances between cognitively healthy controls (CO) and patients diagnosed with Alzheimer’s disease (AD), dementia with Lewy bodies (DLB), frontotemporal dementia (FTD), or Parkinson’s disease (PD). Each point corresponds to a protein, with colored points denoting those with significant abundance differences, identified by an adjusted p-value of < 0.05. The x-axis represents the effect size, while the y-axis depicts the -log10 of the raw p-value, highlighting proteins with both substantial and statistically significant abundance changes. (**E**) The heatmap illustrates the correlation of effect sizes for proteins significantly associated (*FDR* < 0.05, *n* = 91) across various disease types. (**F**) The heatmap displays the correlation of effect sizes for proteins significantly associated with AD (*FDR* < 0.05) and other diseases (*p* < 0.05, *n* = 101) across various disease types. (**G**-**L**) Scatter plots comparing the effect sizes of 123 proteins across different diseases (AD, DLB, FTD, and PD). Red circles indicate proteins that pass the FDR threshold (adjusted p-value < 0.05) from the z-test, with text labels added for the top 20 proteins with the smallest FDR values. Blue squares represent nominally significant proteins (p-value < 0.05 but not FDR-significant), with the top 5 (by smallest p-value) labeled. Grey circles indicate proteins that are not statistically significant

We performed linear regression analyses to identify proteins associated with AD (*n* = 1,123), DLB (*n* = 75), FTD (*n* = 58), and PD (*n* = 126; Table 1). Among the 123 protein analytes measured, 81 were associated with AD, 21 with DLB, four with FTD, and 52 with PD after FDR correction (Fig. 2A–D, Supplementary Table 1 and 2). Three proteins: NEFL, Aβ38 and CCL2, were associated with the four diseases, and 10 proteins (ANXA5, Aβ40, Aβ42, GOT1, NPY, PRDX6, p-tau217, p-tau231, TEK, VEGFD) were associated with DLB, PD and AD. GFAP showed association with AD, DLB and FTD but not PD. (Fig. 2A–D, Supplementary Fig. 1-2).

To characterize both shared and disease-specific alterations, we used two complementary approaches: (1) comparisons of effect sizes across diseases, and (2) a scorecard system ranking proteins by their associations with multiple diseases (see Methods; Supplementary Table 3, Supplementary Fig. 3). These analyses highlight CCL2, NEFL, VEGFD, and GFAP, which clustered together, showed the highest absolute scores, and were all positively associated with all diseases after FDR. Aβ38 also showed one of the highest absolute scores. It was associated with all diseases, after FDR correction, but with a negative effect size. Consequently, it clustered with Aβ40, Aβ42, and TEK, which were also negatively associated with AD, PD, and DLB after FDR correction, and only nominally associated with FTD. A group of six proteins (positive in most association: ANXA5, PRDX6, p-tau217, p-tau231; negative association GOT1 and NPY) that were associated with three of the four diseases were next in ranking (Supplementary Table 3, Supplementary Fig. 3).

P-tau217 was the most significantly associated protein with AD (*β* = 0.996; *FDR-adjusted p* = 1.57 × 10^−186^; Supplementary Table 1). Although it was also significantly associated with DLB (*β* = 0.306; *FDR-adjusted p* = 4.45 × 10^−3^) and PD (*β* = −0.204; *FDR-adjusted p* = 6.96 × 10^−3^), it was not the top hit for these diseases. No significant association was observed between p-tau217 and FTD. Other highly significant proteins for AD included GFAP (*β* = 0.676; *FDR-adjusted p* = 5.02 × 10^−102^), p-tau231 (*β* = 0.700; *FDR-adjusted p* = 4.42 × 10^−94^), NEFL (*β* = 0.417; *FDR-adjusted p* = 1.52 × 10^−48^) and p-tau181 (*β* = 0.483; *FDR-adjusted p* = 9.25 × 10^−46^; Supplementary Table 1). Our analysis also highlighted other well-known AD-associated proteins involved in AD-related pathways, including full-length MAPT, APOE, PSEN1, and BACE1, as well as proteins reported to be associated with AD previously in some large-scale proteomics studies, including NPTXR, ACHE, BDNF, IL7, VEGF-D, and VEGF-A [36–38]. Interestingly, proteins known to be implicated in neurodegenerative diseases other than AD, such as SNCA, pSNCA-129, Oligo-SNCA, SOD1, and TARDBP were also significantly associated with AD. Conversely, some proteins reported to be associated with AD in CSF [38, 39], such as YWHAZ, NPTX1, SMOC1, YKL40 (CHI3L1), did not show significant associations in plasma.

Aβ38 (*β* = −0.369; *FDR-adjusted p* = 8.35 × 10^−3^), CCL2 (*β* = 0.470; *FDR-adjusted p* = 8.35 × 10^−3^), GFAP (*β* = 0.350; *FDR-adjusted p* = 9.68 × 10^−3^), and NEFL (*β* = 0.950; *FDR-adjusted p* = 2.66 × 10^−23^) were associated with FTD after multiple test correction. All four proteins were also associated with DLB and AD, while Aβ38, CCL2, and NEFL additionally showed association with PD. NEFL showed higher effect size in FTD than in other diseases (Supplementary Table 1). We identified 21 proteins associated with DLB after multiple test correction, including MSLN (*β* = 0.292; *FDR-adjusted p* = 3.93 × 10^−2^) and SAA1 (*β* = 0.360; *FDR-adjusted p* = 8.07 × 10^−3^) that were associated exclusively with DLB. Among the 52 PD-associated proteins, five proteins (CCL22, CD63, FCN2, IL10, and IL18) were not linked to any other diseases.

To further evaluate how similar or different AD, FTD, DLB, and PD are, we assessed the correlations of effect sizes across these diseases (Fig. 2E–L). The strongest correlation was observed between PD and DLB (*r* = 0.60, *p* = 2.52 × 10^−9^), followed by AD and DLB (*r* = 0.59, *p* = 9.95 × 10^−10^), indicating a significant overlap in the proteomic profile underlying these two sets of neurodegenerative diseases. The smallest overlap across disease pairs was found between AD and PD (*r* = 0.02, *p* = 0.82).

To determine the proteins driving the high or low cross-diseases correlations, we compared the effect sizes for each individual protein. (Fig. 2G–L). We have previously used a similar approach to compare the proteomic profiles between autosomal-dominant and sporadic AD. [37]When comparing PD and DLB, we identified 24 proteins with discordant effect sizes (Supplementary Table 2, Supplementary Fig. 4), including p-tau217, p-tau231, p-tau181, ACHE, and MAPT that showed opposite direction between DLB (positive) and PD (negative).(Fig. 2L) PARK7 showed positive effect size in PD but no association with DLB, whereas FLT1 shower higher negative effect size in PD than DLB. PARK7 also showed opposite direction in FTD when compared to PD. When comparing AD and PD, PARK7, Oligo-SNCA, SNCA, pSNCA-129, SOD1 and ENO2 showed opposite effect size (Supplementary Table 1). AD with DLB, which was the second disease pair with highest correlation (*r* = 0.59), also included proteins with disease-specific associations. We identified 40 proteins with different effect size between AD and DLB, and 27 proteins such as SAA1, NRGN, TARDBP, SOD1, ARSA or VSLN1 that were in opposite directions and could potentially be used for differential diagnosis (Fig. 2H). AD and FTD also showed relatively high correlation in protein abundance pattern (*r* = 0.42, *p* = 3.95 × 10^−5^) but included several disease-specific proteins as well. Even though NEFL was positively associated with both these diseases, the effect size of NEFL for FTD was higher than in AD. The p-tau proteoforms as well as ACHE, SNAP25, which were highly associated with AD showed almost no association with FTD. Similarly, MME, SQSTM1, TARDBP showed negative effect size for AD but positive for FTD (Supplementary Table 1). When comparing FTD and DLB (*r* = 0.55, *p* = 1.46 × 10^−8^), we also found that SNCA, and Oligo-SNCA had a positive association with DLB but a negative association with FTD, likely capturing synuclein pathology. Taken together, these findings suggest that increased levels of PARK7 is specific to PD, with a significant but negative association in AD. Synuclein-related assays (Oligo-SNCA, SNCA, pSNCA-129) appear to capture synuclein pathology related to PD, whereas NEFL seems to be a better marker for FTD, and p-tau217 for AD.

**Fig. 3:**
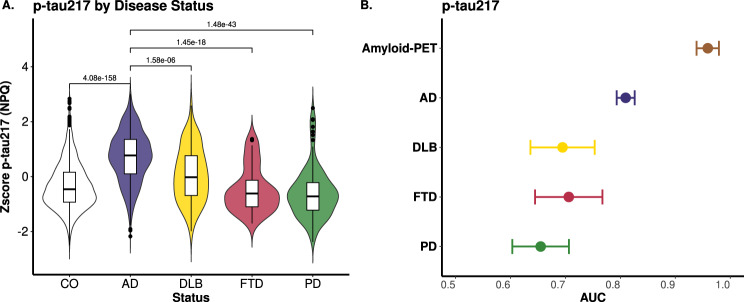
Plasma p-tau217 expression across diseases. (**A**) the violin plot shows the distribution of z-scored log10 p-tau217 (NPQ values) across different clinical statuses, including cognitively healthy controls (CO), Alzheimer’s disease (AD), and other conditions, with significance annotations highlighting statistically significant differences in p-tau217 levels between these conditions. (**B**) The graph illustrates the predictive ability of p-tau217, sex, and age for Amyloid-PET (cutoff = 20) and differentiating AD, DLB, FTD, and PD from controls through logistic regression. It displays the AUC (area under the curve) and confidence intervals for each dataset

We also performed additional sensitivity and specificity analyses across AD and Alzheimer's Disease and Related Dementias (ADRDs) for p-tau217 since it was the protein with the strongest association with clinical AD status. Plasma p-tau217 levels showed significant differences between AD and CO (*p* = 4.08 × 10^−158^), as well as between AD and DLB (*p* = 1.58 × 10^−6^), AD and FTD (*p* = 1.45 × 10^−18^), and AD and PD (*p* = 1.48 × 10^−43^; Fig. 3A). A detailed analysis of the p-tau217 distribution in FTD, DLB, and PD revealed bimodal pattern, with some individuals exhibiting p-tau217 levels close to those in AD. This observation suggests that there is a percentage of DLB, FTD and PD samples with amyloid pathology or a potential misdiagnosis. When we evaluated the predictive power of p-tau217 across these diseases, the AUC for clinical AD was 0.81, which was significantly higher than that for DLB (*AUC* = 0.70), FTD (*AUC* = 0.71) and PD (*AUC* = 0.66) (Fig. 3B).

### AD endophenotypes show different proteomic signatures

Next, we also performed additional analyses for of AD-related phenotypes: Amyloid-PET (*n* = 313), Tau-PET imaging (*n* = 266), CDR (*n* = 2,719) and CSF Aβ42/Aβ40 ratio (*n* = 529, Table 2).

**Fig. 4:**
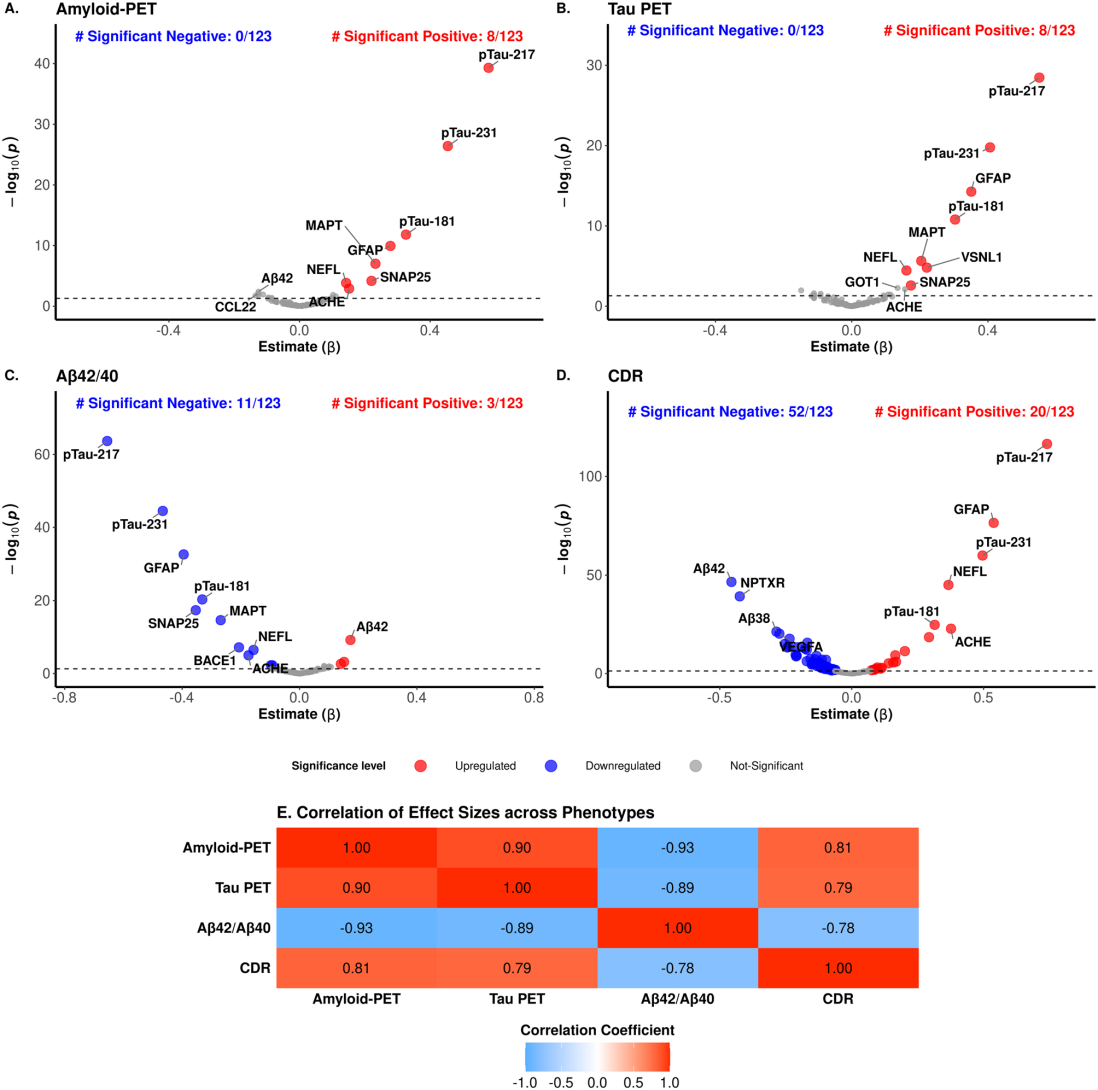
Association of plasma proteins with AD endophenotypes. (**A–D**) The volcano plots display proteins, selected from the panel of 123 proteins, that show significantly different abundances between Alzheimer's disease (AD) and control (CO) groups, stratified by Amyloid-PET, Tau PET, CSF Aβ42/Aβ40 ratio, and CDR levels. Each point corresponds to a protein, with colored points denoting those with significant abundance differences, identified by an adjusted p-value of < 0.05. The x-axis represents the effect size, while the y-axis depicts the -log10 of the raw p-value, highlighting proteins with both substantial and statistically significant abundance changes. (**E**) The corresponding heatmap presents the correlation of effect sizes for these significant proteins across multiple phenotypic measures

We identified eight proteins associated with Amyloid-PET, eight with Tau-PET, 14 proteins with the CSF Aβ42/Aβ40 ratio, and 72 proteins with CDR (Fig. 4A–D, Supplementary Table 4). P-tau217 was the most significant protein for all four phenotypes. Other AD-related markers associated with all four endophenotypes included p-tau231, p-tau181, MAPT, GFAP, SNAP25 and NEFL. Similar to the comparison across the diseases described in the previous section, we assessed the uniqueness of proteomic signatures for each AD phenotype using effect size and protein level correlations. (Fig. 4E, Supplementary Fig. 5, Supplementary Table 5). The strongest absolute correlation was observed between Aβ42/Aβ40 and Amyloid-PET (*r* = −0.93, *p* = 5.20 × 10^−33^; Fig. 4E), suggesting that CSF Aβ42/Aβ40 and Amyloid-PET are capturing similar proteomic signatures. However, there were 28 proteins that showed significantly different effect size between these two phenotypes. All p-tau proteoforms, GFAP, SNAP25, NEFL, ACHE showed higher effect size in Amyloid-PET than Aβ42/Aβ40. MME, IL18, and IL9 showed a higher effect size for CSF Aβ42/Aβ40 than Amyloid-PET or Tau-PET suggesting that these proteins could be a CSF specific biomarker (Supplementary Fig. 5). We also found strong effect size correlations between Tau-PET and Amyloid-PET (*r* = 0.90) and Tau-PET and CSF Aβ42/Aβ40 (*r* = −0.89). The weakest correlation was observed between CDR and Aβ42/Aβ40 (*r* = −0.78) and CDR and Tau-PET (*r* = 0.79; Fig. 4E). The proteins that were driving these lower correlations were ACHE, NPTXR, ARSA, BDNF or TIMP3 among others.

Since p-tau217 was the most significant association in all four phenotypes, we assessed its predictive power for these four endophenotypes. ROC analysis showed that p-tau217 achieved an AUC of 0.81 for AD status and 0.96 for Amyloid-PET (Fig. 3). Then, we assessed if combining p-tau217 with protein ratios from Aβ40, Aβ42, BDNF, and NPTXR, could enhance predictive power. The p-tau217/NPTXR ratio showed the best performance (*AUC* = 0.86; 95% *CI*: 0.84–0.87) for clinical AD status, followed by the p-tau217/Aβ42 ratio (*AUC* = 0.84, 95% *CI:* 0.83–0.86), both besting the standalone p-tau217 model (*AUC* = 0.81; Supplementary Fig. 6). The p-tau217/BDNF ratio showed the poorest performance AUC (0.70, 95% *CI*: 0.68–0.72). For Amyloid-PET classification, standalone p-tau217 achieved the highest AUC (0.96), closely followed by p-tau217/NPTXR and p-tau217/Aβ42 (*AUC* = 0.93 and 0.92; respectively; Supplementary Fig. 6). Overall, we observed that p-tau217 showed strong predictive ability in distinguishing between clinical AD and CO, as well as in classifying amyloid-PET biomarker positive and negative participants. However, combining p-tau217 with other markers enabled a more comprehensive prediction of clinical disease.

### Plasma p-tau217 as a biomarker of brain amyloidosis

Given the strong association between p-tau217 and Amyloid-PET, we evaluated the predictive power of plasma p-tau217 for Amyloid-PET positivity and used multiple approaches to establish potential cutoffs for plasma p-tau217 levels measured using NULISAseq.

**Fig. 5:**
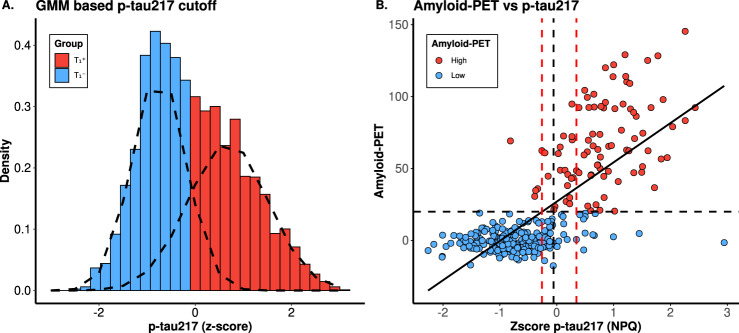
Identification of p-tau217 positivity cut off and its association with amyloid imaging. (**A**) the density plot shows the normal distribution of Z-scores from GMM using p-tau217 data. The x-axis represents Z-scores, with the red part for p-tau-positive samples and the blue part for p-tau-negative samples. The intersection of the colors marks the cutoff value. (**B**) The scatterplot illustrates a positive correlation between p-tau217 and Amyloid-PET. The vertical black dashed line indicates Youden's index single cutoff for plasma p-tau217, which optimally balances sensitivity and specificity in distinguishing high ( > 20) from low ( < 20) amyloid-PET levels. The intermediate range of plasma p-tau217 is shown with the lower and upper vertical red dashed lines, representing the values associated with 95% sensitivity (lower line) and 95% specificity (upper line) for differentiating high ( > 20) from low amyloid-PET levels ( < 20). Out of 325 samples, 52 (16.00%) fall within the plasma p-tau217 intermediate value range

We used a data driven model to identify the cut-off for biomarker positivity, as described in previous studies [28, 40–42]. Specifically, we used GMM, including data from all samples with p-tau217 values (*n* = 3,938) to identify the cut-off. Through this approach, we established the p-tau217 z-score cutoff at −0.06, which corresponds to a linear NPQ value of 4574.13 (Fig. 5A). Based on this cutoff, 2,109 samples were classified as biomarker-negative (T_1_^-^), while 1,829 were classified as biomarker-positive (T_1_^+^). The Youden Index [43] was also used to determine a single cutoff, which matched GMM-derived value when rounded to two decimal places.

A confusion matrix was constructed from samples with both p-tau217 levels and amyloid-PET information (*n* = 325) to assess the p-tau217 cutoff concordance with Amyloid-PET positivity. The overall concordance rate between p-tau217 and amyloid-PET-based biomarker status was 91.38% (28 misclassified participants), and in good agreement with the literature reported value of 90% (Fig. 5B, Supplementary Table 6) [43]. This analysis also yielded an AUC of 0.96 for plasma p-tau217 vs. Amyloid-PET. (*PPV*: 0.82 and *NPV*: 0.96; Supplementary Table 7).

Previous research has suggested that a two-cutoff approach, which includes an intermediate class of samples, outperforms a single-cutoff approach [43]. We tested this by using p-tau217 levels at 95% sensitivity and 95% specificity as the lower and upper cutoffs. Samples were categorized into low (*n* = 79, 24.31%; z-score p-tau217 < −0.26), intermediate (*n* = 52, 16.00%; z-score p-tau217 = −0.26–0.35), and high (*n* = 194, 59.69%; z-score p-tau217 > 0.35). We then checked concordance by only including the high and low classes. The concordance rate improved from 91.38% in the single cutoff classification to 93.77% in the double cutoff approach (Supplementary Table 6).

We also evaluated the correlation between plasma p-tau217 and Tau-PET, observing a moderate association between the two (*r* = 0.39, *p* = 2.70 × 10^−10^; Supplementary Fig. 7A-D). Applying the same two-threshold cutoff approach yielded a 93.09% concordance of p-tau217 with and Tau-PET (Supplementary Fig. 7A; Supplementary Table 6) and an AUC of 0.93 when adjusted with age and sex (95% *CI*: 0.89–0.98), which is lower than that for Amyloid-PET (Supplementary Fig. 7B, 7C, Supplementary Table 7). Without adjusting for age and sex, the AUC for Tauopathy is 0.93, which is slightly lower than that for Amyloid-PET (*AUC* = 0.96; Supplementary Fig. 7D).

Finally, we examined whether the correlation between p-tau217 and Tau-PET results was influenced by all individuals or primarily by those who were amyloid or Tau-PET positive. The analysis revealed that the correlations were predominantly driven by biomarker-positive individuals (*r*_*Amyloid*_ = 0.43, *r*_*Tau PET*_ = 0.51), rather than biomarker-negative individuals (*r*_*Amyloid*_ = 0.17, *r*_*Tau PET*_ = 0.03; Supplementary Fig. 8A-F).

### Identifying proteins associated with progression to symptomatic AD

**Fig. 6:**
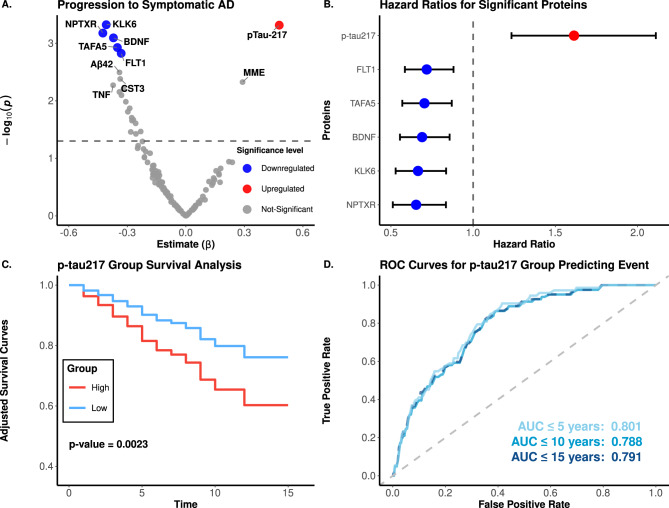
Proteins associated with progression to symptomatic AD. (**A**) differential abundance analysis compares individuals initially classified as CO who either converted to AD or remained CO. In the volcano plot, colored dots indicate p-adjusted values < 0.05. (**B**) Whisker plot of hazard ratios and 95% confidence intervals for all significant proteins in panel A. (**C**) Kaplan-Meier survival curves show the progression to AD following the initial blood draw. The biomarker-low group (blue line) and the biomarker-high group (red line) illustrate the proportion of participants who remain cognitively normal over the years of follow-up. The p-value highlights the significant difference in AD progression between individuals predicted to have AD and controls, based on the prediction model. (**D**) Receiver operating characteristic (ROC) curves assessing the predictive power of p-tau217 in classifying clinical Alzheimer’s disease (AD) over 5, 10, and 15 years

Among the 1,596 CO participants at the time of plasma collection, 952 underwent follow-up clinical assessments in AD research. Of these, 84 progressed to symptomatic AD over a follow-up period of one to 23 years. We used these data to identify proteins associated with AD progression. Using a Cox proportional hazards model, adjusted for age at blood draw and sex, we identified six proteins that were associated with disease progression (Fig. 6). BDNF (*hazard ratio* [*HR*] = 0.69, *FDR-adjusted p* = 2.47 × 10^−2^), KLK6 (*HR* = 0.67, *FDR-adjusted p* = 2.47 × 10^−2^), NPTXR (*HR* = 0.65, *FDR-adjusted p* = 2.47 × 10^−2^), TAFA5 (*HR* = 0.70, *FDR-adjusted p* = 2.92 × 10^−2^), and FLT1 (*HR* = 0.72, *FDR-adjusted p* = 3.07 × 10^−2^, Fig. 6A–B, Supplementary Table 8) exhibited significant protective associations, indicating their potential roles in reducing the risk of developing symptomatic AD when accumulated.

In contrast, higher p-tau217 levels were associated with an increased risk of progression to symptomatic AD (*HR* = 1.61, *FDR-adjusted p =* 2.47 × 10^−2^, Fig. 6A–B). Over a 15-year period, subjects with higher p-tau217 levels were more likely to progress to AD compared to subjects with lower p-tau217, more than 70% of whom remained disease-free within the timeframe (*p* < 0.01, Fig. 6C) with an AUC of 0.801 when predicting subjects that progressed to AD within 5 years (Fig. 6D). For longer durations, the predictive power for those progressing to symptomatic AD at 10 and 15 years was 0.788 and 0.791, respectively (Fig. 6D). Together, these findings underscore the potential of p-tau217 in monitoring disease and predicting symptom onset in pre-symptomatic individuals.

### Pathway analyses highlight shared and disease-specific pathways

**Fig. 7:**
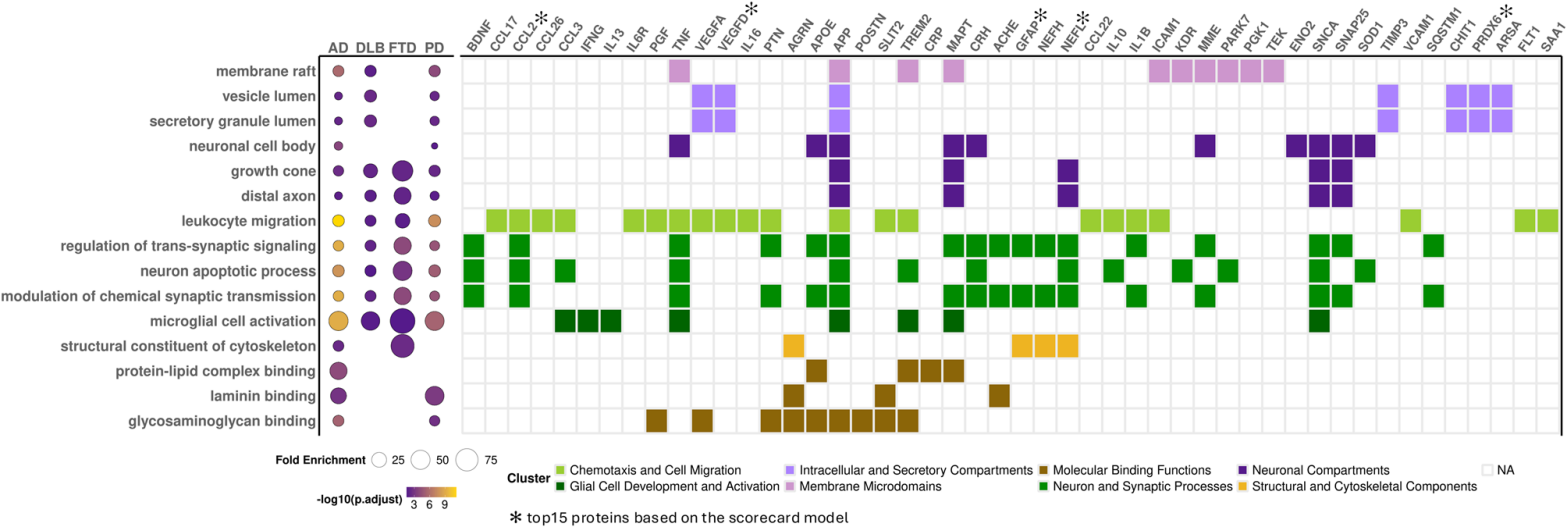
Disease-specific pathway analysis. Enriched pathways across diseases (AD, DLB, FTD, and PD), where each point represents a pathway–disease pair. Point size indicates Fold enrichment, and fill color represents statistical significance (−log10 FDR), with brighter colors indicating higher significance. (right) This heatmap shows the presence of specific genes (x-axis) across enriched biological pathways (y-axis). Colored tiles indicate pathway–gene associations observed in at least one disease, grouped into functional clusters (e.g., neuronal compartments, binding functions). Each color corresponds to a distinct biological cluster, as defined in the figure legend below the plot

We performed pathway enrichment analysis using the proteins associated with the four studied disease. Overall, pathway analysis indicated that these proteins are primarily associated with eight central mechanisms in all neurodegenerative diseases:1) Molecular Binding Functions, 2) Structural and Cytoskeletal Components, 3) Glial Cell Development and Activation, 4) Neuron and Synaptic Processes, 5) Chemotaxis and Cell Migration, 6) Neuronal Compartments, 7) Intracellular and Secretory Compartments, and 8) Membrane Microdomains (Fig. 7; Supplementary Fig. 9; Supplementary Table 9).

The main sub-pathway for the Membrane Microdomains was membrane raft (*FDR-adjusted p* < 1.70 × 10^−2^; Supplementary Table 9), that included known proteins implicated in AD (APP, TREM2), PD (PARK7), and FTD (MAPT), as well as novel proteins identified in this study related to endothelial health ICAM1, TNF, KDR among others (Supplementary Table 9).

Intracellular and Secretory Compartments included several major sub-pathways such as vesicle lumen (*FDR-adjusted p* < 8.26 × 10^−3^), and secretory granule lumen (*FDR-adjusted p* < 8.26 × 10^−3^; Supplementary Table 9). Key proteins identified in these pathways were vascular factors (VEGFD, VEGFA) and others (APP, TIMP3, CHIT1, PRDX6, and ARSA). Secretory granule lumen appeared to be more enriched in DLB. (Supplementary Table 9)

Neuronal and microglia-related pathways were shared across all diseases. These included two major neuronal pathways related to neuronal compartments and neuron and synaptic functions. Even though the overall pathways were shared, they appeared to be driven by different proteins in each disease. For example, the neuronal compartments pathway association was driven by APP and MAPT for AD but SNCA for PD. Multiple sub pathways were identified for synaptic functions driven by a set core of proteins associated with all diseases (BDNF, CCL2, ACHE, GFAP, NEFH or NEFL) among others. The microglia cell activation pathway, also shared across all diseases, was mainly driven by TREM2, a known microglia protein, along with IL13, IL6R and IFNG.

In addition to the pathways described above, we also identified several disease-enriched pathways based on fold enrichment analysis. The protein-lipid complex binding pathway (*p* = 9.52 × 10^−5^) was distinctive to AD and predominantly driven by APOE, TREM2, CRP and MAPT (Supplementary Fig. 9). Structural constituent of cytoskeleton is more enriched in FTD and driven by GFAP and NEFL. PD-associated proteins were enriched on the laminin binding (*p* < 1.72 × 10^−3^), primarily driven by AGRN, SLIT2 and ACHE (Supplementary Fig. 9, 10).

In summary, major pathways previously implicated in neurodegeneration were replicated in our study and were driven by the most significant proteins from the abundance analysis.

### Technical properties and orthogonal validation of NULISA assays

First, we assessed the reproducibility of NULISA measures by measuring the same set of samples in two separate experiments on different dates. (*n* = 86, Supplementary Table 10). The average correlation observed was 0.75 (Supplementary Fig. 11) with 69.92% (86 out of 123) analytes showing a correlation higher than 0.7 (Supplementary Table 11). Neurofilament Heavy Chain (NEFH) showed the highest reproducibility with correlation between experiments being 0.98 (*p* = 5.28 × 10^−56^). Other proteins that demonstrated high reproducibility included NRGN (*r* = 0.98, *p* = 4.15 × 10^−56^), FGF2 (*r* = 0.97, *p* = 7.73 × 10^−54^), GDNF (*r* = 0.97, *p* = 1.47 × 10^−51^), MME (*r* = 0.97, *p* = 1.93 × 10^−52^) and ANXA5 (*r* = 0.97, *p* = 7.45 × 10^−52^; Supplementary Table 11). The AD/ADRD-related biomarkers such as p-tau217, p-tau181, p-tau231, TREM2, GFAP, NRGN, and NEFL all exhibited correlations greater than 0.87 (Supplementary Table 11, 12).

We found that proteins with high correlation across experiments also exhibited higher correlation in different matrices (Supplementary Fig. 11). Further analysis showed that proteins with higher correlations across experiments had higher IQRs, while those with lower correlations had lower IQRs (Supplementary Fig. 11C; Supplementary Table 13, 14). These findings suggest that proteins demonstrating consistent reproducibility also exhibit high biological variability.

EDTA is commonly used for hematological exams, with many blood samples anticoagulated with it. Sodium citrate is preferred for coagulation studies due to its reversible effect with Ca2+ addition [44]. In this study, we measured 43 samples using the NULISA CNS panel with two sample stabilization additives, EDTA and sodium citrate (*n* = 43; Supplementary Table 10). We found an average correlation of 0.67 between the preservation agents, with 53.66% (66 out of 123) analytes with correlation of 0.70 or higher (Supplementary Fig. 11D; Supplementary Table 11). The highest correlation was observed for NEFH (*r* = 0.99, *p* = 5.21 × 10^−37^) followed by GDNF (*r* = 0.99, *p* = 1.28 × 10^−31^, Supplementary Table 11). The AD/ADRD biomarkers p-tau217, p-tau181, p-tau231, TREM2, GFAP, and NEFL all had correlations > 0.84. Oligo-SNCA showed a negative correlation (*r* = −0.05), but it was not statistically significant. (Supplementary Table 11).

To further perform technical validation of the NULISA assay, we leveraged proteomic data for samples measured using immunoassay techniques (*n* = 1,466) and SomaScan (*n* = 3,716; Supplementary Table 15). Immunoassay measurement was available for p-tau181, Aβ40, Aβ42, GFAP, NEFL. We observed high correlation for GFAP (*r* = 0.83, *p* = 3.4 × 10^−151^), NEFL (*r* = 0.81, *p* = 1.2 × 10^−138^), and p-tau181 (*r* = 0.71, *p* = 1.6 × 10^−95^) between NULISA and immunoassay measurements (Supplementary Fig. 12) as previously reported [15]. Moderate correlation for Aβ40 (*r* = 0.63, *p* = 1.1 × 10^−164^) and Aβ42 (*r* = 0.51, *p* = 3.2 × 10^−96^) was observed.

Of the 123 NULISA CNS proteins, 112 are also included in the SomaLogic 7K panel (148 total analytes pairs, as SomaLogic has more than one assay for the same proteins). Overall, 125 analytes showed a significant correlation (*p* < 0.05; Supplementary Table 16), and the mean correlation between the platforms was 0.29 (Supplementary Fig. 13; Supplementary Table 16). Eight proteins pairs showed a correlation higher than 0.8, and 23 proteins pairs higher than 0.7. The highest correlation was observed for CHIT1(analyte X3600.2; *r* = 0.92, *p* < 10^−300^) followed by TREM2 (analyte X5635.66; *r* = 0.87, *p* < 10^−300^; Supplementary Fig. 13). Other known markers of neurodegeneration NRGN and CRP showed correlations of 0.75 (*p* < 10^−300^) and 0.82 (*p* < 10^−300^) respectively (Supplementary Table 16, Supplementary Fig. 13).

Overall, we observed that the majority of NULISA assays produced reliable results across experiments, with assay-dependent high correlation identified between NULISA and other proteomic platforms.

### NULISAseq classification of *APOE* genetic status

The *APOE ε4* allele is the most significant genetic risk factor for AD; a single ε4 allele increases disease risk by two to four fold [45]. An early determination of *APOE ε4* carrier status can help AD risk management through targeted therapies and lifestyle changes. NULISAseq CNS Panel includes an assay targeting the APOE ε4 proteoform, informing on whether an individual expresses the ε4 allele. Of the 3,947 samples measured using the NULISAseq platform, *APOE* genotype information was available in 3,892 samples through direct SNP genotyping (Taqman). The presence or absence of variants rs423958 and rs7412 was used to infer *APOE* status. Individuals with genotype of ε2/ε2, ε2/ε3, ε3/ε3 alleles were labelled as *APOE4-* and those who had ε2/ε4, ε3/ε4 or ε4/ε4 genotype were considered *APOE4+.*

We observed a concordance rate of 99.00% between NULISAseq and genotyped-based *APOE* status assignment (Supplementary Table 17). Among the 1,573 samples assigned as *APOE4+* through genotyping, NULISAseq confirmed that 1,549 expressed the ε4 isoform, while remaining 24 samples were classified as *APOE4-.* Of the 232 samples genotyped as homozygous ε4, NULISAseq labeled all but three as *APOE4+*, achieving 98.71% concordance within the group (Supplementary Table 18). Participants who were homozygous ε3 carriers accounted for 14 of the 39 total mismatches. Similarly, NULISAseq correctly classified 99.35% (2,304 of 2,319) of *APOE4-* individuals, as determined through genotyping, as lacking the ε4 isoform. Of the 15 samples incorrectly labeled as *APOE4+,* one was ε2/ε3 heterozygous, and 14 were homozygous ε3. Despite correctly labeling 99.03% (3,486 of 3,520) of ε3, most of the discordance was driven by samples carrying at least one ε3 allele (15 genetic *APOE4-* labelled as *APOE4+* and 19 genetic *APOE4+* labelled as *APOE4-*). Overall, these results demonstrated a robust concordance of classifying *APOE ε4* status between genotyping and NULISAseq.

## Discussion

In recent decades, significant progress has been made in discovering novel biomarkers of AD and ADRDs [7, 46, 47]. Blood-based biomarkers are attractive due to the simplicity of sample collection and the potential for widespread adoption [48–50]. The advent of sensitive, multiplexed, and indirect measurement proteomic platforms has further enhanced the potential utility of these fluid biomarkers. Larger studies interrogating the accuracy, sensitivity, and specificity of these assays are necessary to validate and instill confidence in their readouts. In this context, our study has evaluated the recently developed multiplex NULISA technology [15]. We analyzed 123 CNS-specific proteins in 3,947 plasma samples (3,232 unique participants), marking a threefold increase in sample size compared to previous studies. [50–52] The study included samples from participants with ADRDs (PD, DLB, FTD, and AD) and cognitively normal controls. Participants were well-characterized, with AD-related phenotypic measurements such as Amyloid-PET, Tau PET, Aβ42/Aβ40 ratio, and CDR. Finally, we compared the protein measurements from this platform with legacy measures such as single-plex immunoassay and an orthogonal multiplex platform, i.e., SomaScan.[33]

Plasma p-tau217, GFAP, NEFL and FLT1 were identified as the most significantly associated proteins in AD, DLB, FTD, and PD, respectively, compared to cognitively normal individuals. (Fig. 2). As p-tau217 was the most significant AD-associated protein, we analyzed its biomarker performance in the context of AD-related phenotypes such as Amyloid-PET, Tau-PET, Aβ42/40 ratio and CDR. The direction of the association was consistent with known AD pathophysiology, with an increased plasma p-tau217 associated with higher amyloid-PET, Tau-PET, and CDR values and a lower Aβ42/Aβ40 ratio. [53–56] Plasma p-tau217 showed very high predictive power for clinical AD status (*AUC* = 0.81) and Amyloid-PET positivity (*AUC* = 0.96), consistent with previous reports that p-tau217 describes amyloid pathology. [57] We also analyzed its predictive power for amyloid and Tau-PET and observed similar AUC for both traces, with a slightly higher AUC for Amyloid (*AUC* = 0.96) than Tau-PET (0.93; Supplementary Fig. 7). In general, our findings are in line with what has been reported in recent studies and highlight the utility of plasma p-tau217 in predicting AD pathology, particularly those characterized by amyloid changes [58, 59].

As the largest study using the NULISAseq CNS Disease Panel till date, we determined the biomarker positivity cutoff for plasma p-tau217 through multiple data-driven methods. Single-threshold cutoff approaches, utilizing the Youden Index or GMM (cutoff = −0.06; linear NPQ = 4574.13), showed high concordance (91.38%) with Amyloid-PET, consistent with findings from a recent study [43]. Applying a two-cutoff approach further enhanced p-tau217‘s predictive performance and improved alignment with Amyloid-PET classifications in overlapping samples. Given the high cost and specialized nature of PET imaging required to determine amyloid-PET values, plasma p-tau217 could be a cost-effective pre-screening tool for imaging analyses.

In our study, p-tau217 was also associated with an increased risk of progressing to AD. In fact, individuals with higher levels of p-tau217 were more likely to progress to AD within 15 years compared to those with lower levels. In contrast, five proteins (NPTXR, KLK6, BDNF, TAFA5 and FLT1) were associated with decreased risk of developing disease and overall were negatively associated in AD subjects compared to cognitive normal individual (Fig. 6, Supplementary Table 1). Previous studies have shown that NPTXR levels are reduced in the CSF and plasma of individuals with AD compared to cognitively normal individuals [60, 61]. NPTXR is a protein primarily found in neurons that plays a key role in synapse organization, and altered levels can also be detected in plasma, making it a potential biomarker for distinguishing AD from controls, as demonstrated in a recent study [36]. KLK6 is involved in the regulation of neuronal function and the breakdown of amyloid-beta plaques in Alzheimer's disease [36, 40]. Our results closely align with a previous study in over 600 individuals with mixed dementia diagnoses and controls that reported plasma neurosin (Kallikrein 6, hk6) levels increase with age in healthy individuals but decrease in patients with AD. [62] Similarly, a recent NULISA-based study found increased plasma KLK6 was linked with a slower rate of neurodegeneration in a preclinical AD cohort [16]. Conversely, increased plasma KLK6 levels have also been reported in patients with more advanced stages of AD, which we did not investigate. [63] Overall, our findings highlight proteins that may exhibit protective effects against AD.

We also observed a clear bi-normal distribution of p-tau217 levels in DLB, FTD and PD (Fig. 3), overlapping with that in controls and some AD cases. This binomial distribution is more accentuated in DLB, which is likely capturing the Aβ pathology also found in DLB cases. [64–67] It is important to consider that individuals with FTD or PD may also exhibit amyloid positivity [68], which is common at the population level in these diseases, even if AD is not the primary driver of their dementia. This amyloid pathology could help explain the observed high p-tau217 levels in these cases, further supporting the notion of pathological overlap.

In AD, besides p-tau217, there were 80 other associated proteins. These included known AD and neurodegenerative biomarkers such as p-tau231, GFAP, Aβ42, NEFL, NPTXR among others. GFAP, which is a marker of reactive astrogliosis, and NEFL, which is a marker of neuroaxonal damage, were the only proteins also associated with DLB and FTD [69]. Both markers have been previously reported to have an exceptional performance in monitoring cognitive changes in DLB [69]. A previous study highlighted the importance of clinical history and visuospatial function assessment, rather than spontaneous extrapyramidal signs (EPS), in differentiating DLB from AD in early stages [70]. While their approach focused on clinical features, our proteomic analysis revealed that AD and DLB share highly correlated profiles (*r* = 0.59) and similar dysregulated pathways, such as microglial activation (TREM2, IL13, IL6R and IFNG) and neuronal processes. Concurrently, proteins such as SAA1, NRGN, TARDBP, SOD1, ARSA or VSLN1 exhibited opposite directions between the diseases, offering potential for differential diagnosis. When extending these analyses and comparison across all the diseases, we found similar patterns: a large overlap and concordance across diseases, yet with distinct disease-specific proteins being evident. For example, PARK7 showed a positive association that was specific to PD. PARK7 is a well know mediator of PD pathogenesis via mitochondrial dysfunction. Mutations in PARK7 have been linked to familial forms of PD [71].

Pathway analysis identified processes associated with neuronal and glia function, cytoskeleton, cellular trafficking and molecular binding, microglia activity. These pathways were, in general, shared across all neurodegenerative diseases, even though the major overlap was found for the synaptic functions (BDNF, CCL2, ACHE, GFAP, NEFH or NEFL) and microglia pathways (TREM2, IL13, IL6R IFNG). The protein-lipid complex binding pathway was unique to AD whereas pathway associated with structural constituent of cytoskeleton were enriched in FTD, and laminin binding pathway in PD pointing out to both a core set of proteins and pathways common across diseases but also unique features for each disease. These unique features could potentially be used for differential diagnosis, to quantify co-pathology, and to understand the distinct biological processes involved in these diseases.

To technically validate the performance of the NULISA platform, we compared analyte measurements from orthogonal protein readouts, inter-day variations, and among plasma stabilization conditions, as well as *APOE4* genetic status. NULISA achieved an average correlation of 0.70 across the five plasma proteins p-tau181, Aβ40, Aβ42, GFAP, NEFL measured with immunoassays. Established markers of Alzheimer’s disease and neurodegeneration, including TREM2, SAA1, CHIT1, CRP and PDGFRB demonstrated strong correlations (*r* > 0.8) in the comparison with SomaLogic measurements. (Supplementary Table 16). Comparison between experiments within NULISA platforms showed that GFAP, p-tau217, p-tau181, NEFH, and NRGN were highly correlated (*r* > 0.9; Supplementary Table 12). We observed that protein measure reproducibility was directly proportional to its IQR (Supplementary Fig. 11). Given that this analysis involved a test–retest of identical samples, we interpret the high variability in certain proteins as likely stemming from low abundance or technical limitations in protein extraction and measurement, rather than true biological differences between subjects. Finally, NULISAseq correctly predicted the APOE4 genetic status of 99.00% of individuals, for whom allele data were available, whereby carriers of at least one ε3 allele accounted for most of the missed classifications. (Supplementary Table 17, 18) These findings suggest that the multiplex assays in the NULISAseq CNS Disease Panel are accurate, consistent, and sensitive to biological variability, making them ideal for blood collection-based disease monitoring and research settings.

Despite being the largest study to date that comprehensively evaluates the biological and technical aspect of the NULISA platform, this study has some limitations. First, while the study included four neurodegenerative diseases, the sample sizes for FTD and DLB were relatively limited. Additionally, the highly heterogeneous nature of FTD may have impacted the uniformity of some signals, further emphasizing the need for larger sample sizes in future studies. Second, the current study utilizes racially homogenous samples with majority being non-Hispanic white. Follow-up studies with ethnic diversity are needed not only to assess plasma proteomic composition differences, but also to validate our findings. Third, since we utilized cross-sectional samples for our analysis, long term changes, trajectories, and stability of the biomarkers will need to be studied in longitudinal studies. Fourth, although this panel included high-relevant proteins that capture AD (p-tau217, p-tau181, p-tau231, Aβ40), synuclein pathologies (SNCA, Oligo-SNCA, pSNCA-129, SNCB, TDP43) and general neurodegeneration (NEFL, NRGN, GFAP, TREM2), it is not clear if these 120+ proteins can capture all the pathways and features implicated on these complex diseases. Therefore, further studies with even larger samples sizes and using high-throughput proteomic assays such as SomaLogic or Olink, similar to those recently published [36, 38, 72, 73] are needed to fully capture all the proteins and pathways, whether shared or disease-specific, implicated on AD, PD, DLB and FTD. Finally, our comparison of NULISA with other established proteomic platforms was constrained to the analytes measured using immunoassays or SomaLogic. Including additional neurodegeneration-related analytes will enhance the comprehensive evaluation of the platform's accuracy.

Overall, our study highlights disease-specific and shared plasma proteomic signatures among neurodegenerative dementias. We provided for the first time a cut off for plasma p-tau217 based on the NULISA assay and confirm that this assay is a strong biomarker of amyloidosis, with a robust correlation to Amyloid-PET levels, showing similar AUCs and amyloidpositive concordance to other assays. These findings further cement the utility of multiplexed NULISA targeted protein quantification, particularly p-tau217 in neurodegenerative disease research and clinical applications.

## Electronic supplementary material

Below is the link to the electronic supplementary material.

Supplementary Material 1

Supplementary Material 2

## Data Availability

Individual-level data for the following cohorts can be requested through these resources:Knight-ADRC: CSF & plasma proteomic & genomic data (https://knightadrc.wustl.edu/data-request-form/)

## Glossary

Aβ: β-amyloid
AD: Alzheimer disease
ADRD: Alzheimer’s Disease and Related Dementias
AUC: Area Under the ROC curve
CDR: Clinical Dementia Rating
DLB: Dementia with Lewy Bodies
FDR: False Discovery Rate
FTD: Frontotemporal Dementia
HR: Hazard Ratio
IQR: Interquartile range
NPQ: NULISA Protein Quantification
NPV: Negative predictive values
PD: Parkinson’s disease
PiB: Pittsburgh Compound-B
PPV: Positive predictive values
p-tau: Phosphorylated tau
ROC: Receiver operating characteristic
SV: Surrogate variable
