# Supplementary material for: High-sensitivity plasma proteomics reveals disease-specific signatures and predictive biomarkers of Alzheimer’s disease phenotypes in a large mixed-dementia cohort
Source: Mol Neurodegener. 2025 Nov 17;20:120. doi: 10.1186/s13024-025-00909-x (PMC12625197; doi:10.1186/s13024-025-00909-x)
Supplement: Supplementary file 1 — Supplementary Material 1 [file 13024_2025_909_MOESM1_ESM.pdf]

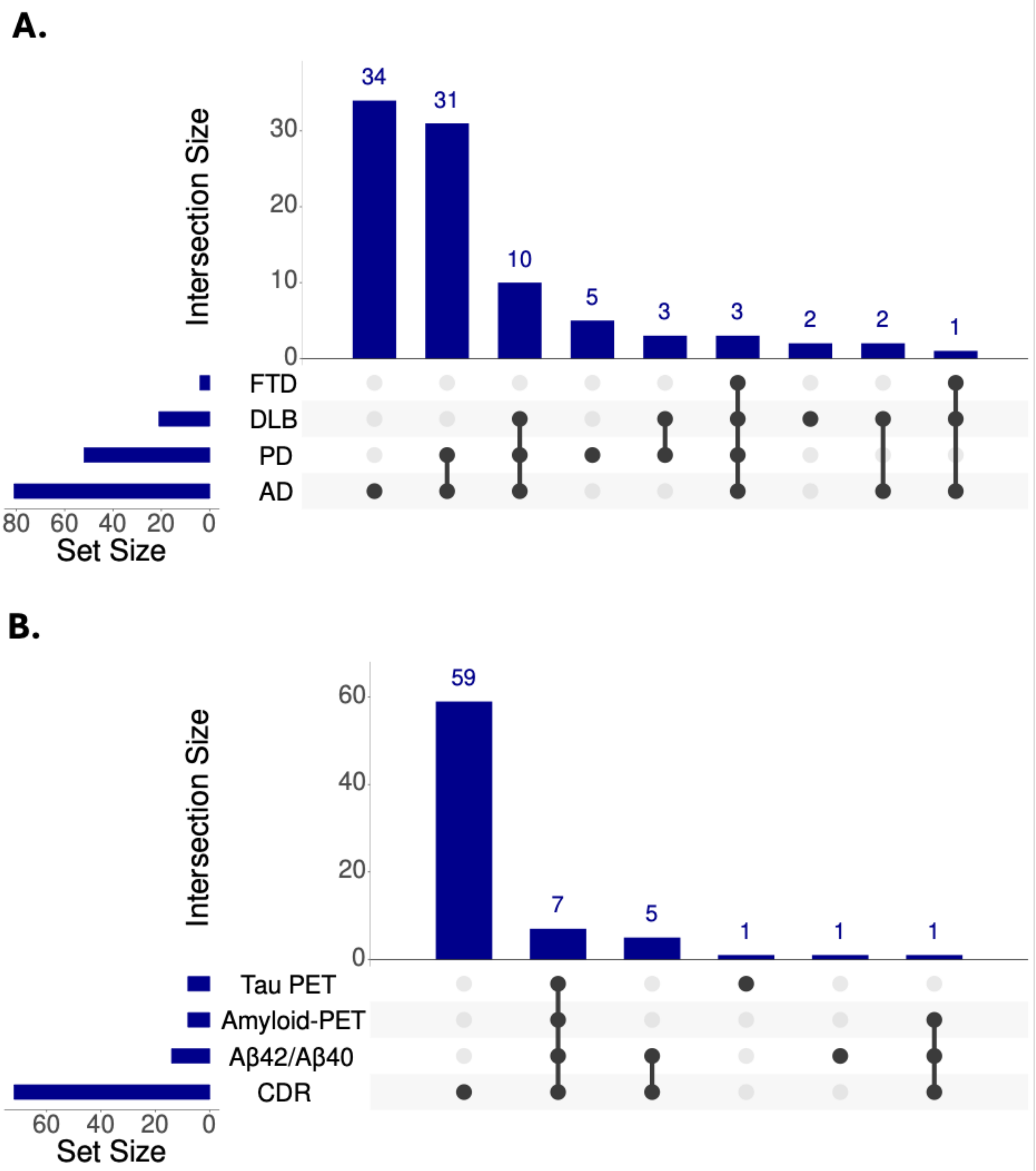

Supplementary Figure 1. Upset Plot Analysis of Shared Proteins in Disease and AD phenotypes. (A) The upset plot demonstrates the overlap of significant analytes (*FDR* < 0.05) across various disease datasets, specifically AD, DLB, FTD, PD. This visualization reveals 91 significant proteins that are identified across these neurodegenerative conditions. **(B)** The upset plot illustrates the overlap of significant analytes (*FDR* < 0.05) associated with the phenotypes Amyloid-PET, Tau PET, Aβ42/Aβ40, and Clinical Dementia Rating (CDR). This analysis identifies a total of 74 significant proteins through differential abundance analysis.

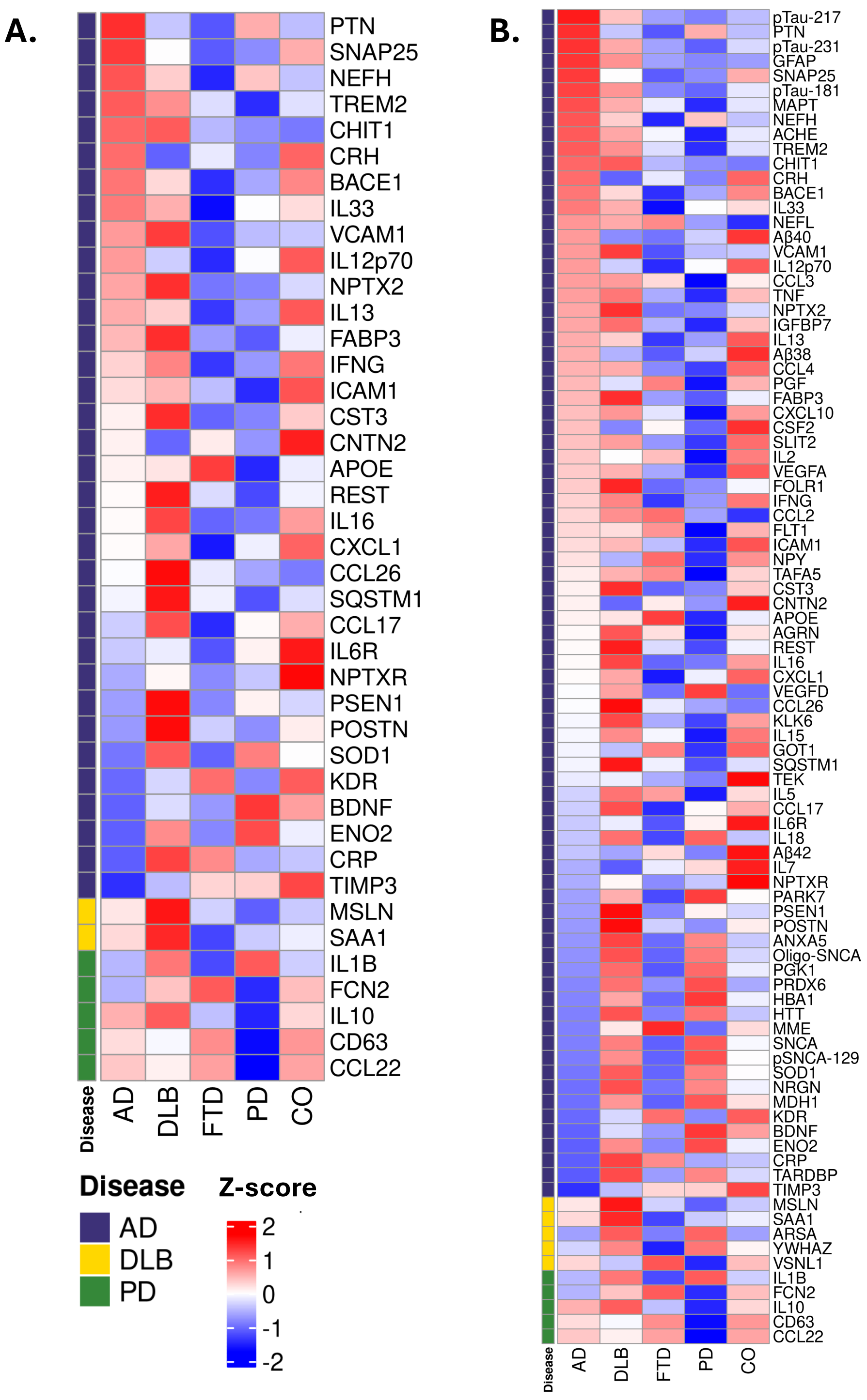

Supplementary Figure 2. Plasma Protein Distribution Across Single and Multiple Diseases. (A-B) We selected proteins that passed the FDR threshold in AD, DLB, FTD, and PD. Plasma protein levels (n = 41 for panel A) were averaged across individuals within each disease (columns) and then normalized across diseases by scaling each protein's values. None were exclusively abundant in FTD, while the proteins (n = 91) in panel B may be abundant across multiple diseases. Proteins abundant in more than one disease were assigned to the first disease they appeared in, based on alphabetical order.

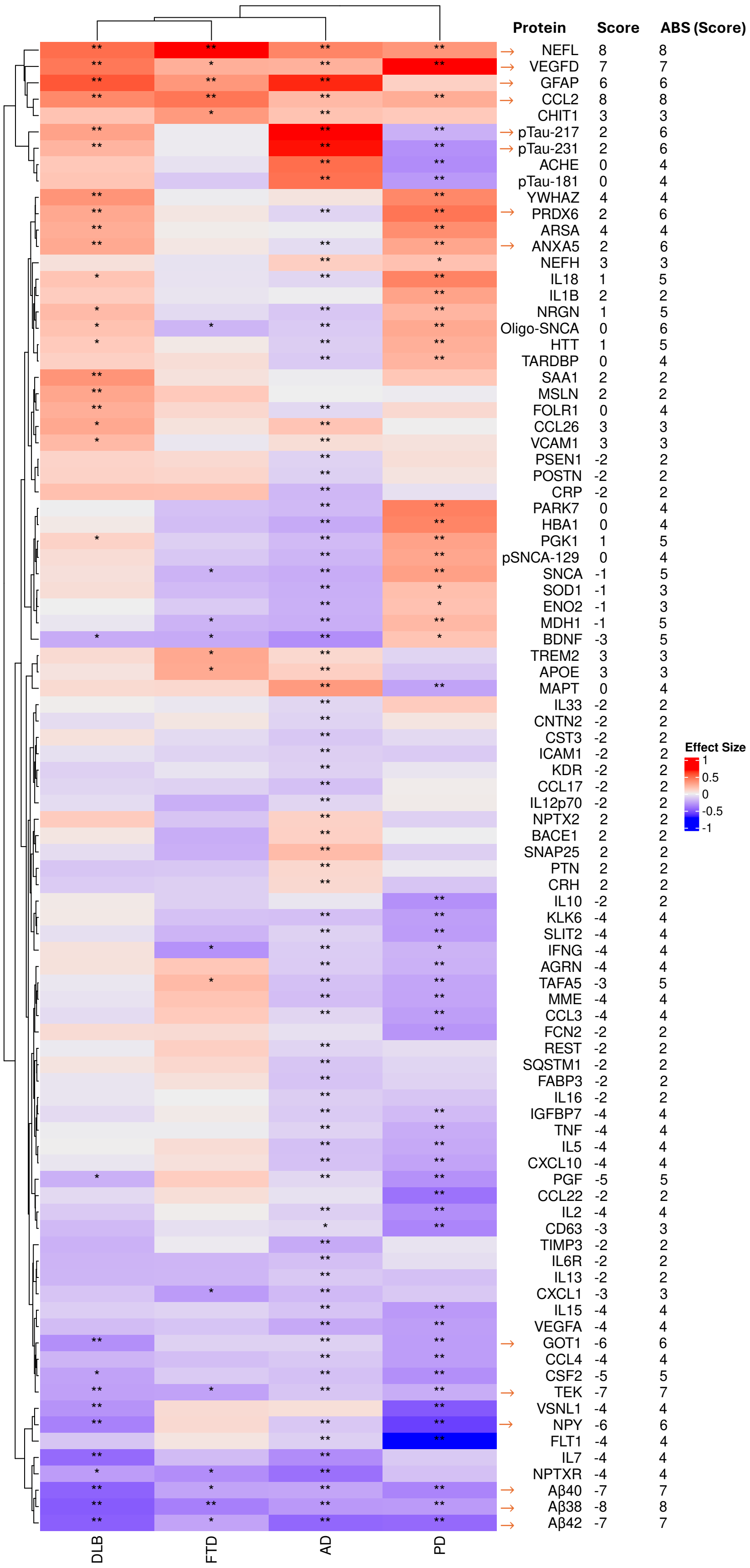
Protein
Score
ABS (Score)
NEFL 8 8
VEGFD 7 7
GFAP 6 6
CCL2 8 8
CHIT1 3 3
pTau-217 2 6
pTau-231 2 6
ACHE 0 4
pTau-181 0 4
YWHAZ 4 4
PRDX6 2 6
ARSA 4 4
ANXA5 2 6
NEFH 3 3
IL18 1 5
IL1B 2 2
NRGN 1 5
Oligo-SNCA 0 6
HTT 1 5
TARDBP 0 4
SAA1 2 2
MSLN 2 2
FOLR1 0 4
CCL26 3 3
VCAM1 3 3
PSEN1 -2 2
POSTN -2 2
CRP -2 2
PARK7 0 4
HBA1 0 4
PGK1 1 5
pSNCA-129 0 4
SNCA -1 5
SOD1 -1 3
ENO2 -1 3
MDH1 -1 5
BDNF -3 5
TREM2 3 3
APOE 3 3
MAPT 0 4
IL33 -2 2
CNTN2 -2 2
CST3 -2 2
ICAM1 -2 2
KDR -2 2
CCL17 -2 2
IL12p70 -2 2
NPTX2 2 2
BACE1 2 2
SNAP25 2 2
PTN 2 2
CRH 2 2
IL10 -2 2
KLK6 -4 4
SLIT2 -4 4
IFNG -4 4
AGRN -4 4
TAFA5 -3 5
MME -4 4
CCL3 -4 4
FCN2 -2 2
REST -2 2
SQSTM1 -2 2
FABP3 -2 2
IL16 -2 2
IGFBP7 -4 4
TNF -4 4
IL5 -4 4
CXCL10 -4 4
PGF -5 5
CCL22 -2 2
IL2 -4 4
CD63 -3 3
TIMP3 -2 2
IL6R -2 2
IL13 -2 2
CXCL1 -3 3
IL15 -4 4
VEGFA -4 4
GOT1 -6 6
CCL4 -4 4
CSF2 -5 5
TEK -7 7
VSNL1 -4 4
NPY -6 6
FLT1 -4 4
IL7 -4 4
NPTXR -4 4
Aβ40 -7 7
Aβ38 -8 8
Aβ42 -7 7
DLB
FTD
AD
PD
Effect Size
1
0.5
0
-0.5
-1

Supplementary Figure 3. Heatmap of protein effect estimates across diseases. We selected 91 proteins that were significant in at least one of the four diseases and visualized their effect estimates in a heatmap, where rows represent proteins and columns represent diseases (AD, DLB, FTD, PD). Colors indicate estimates, with blue representing negative effects, white representing no effect, and red representing positive effects. Significance in each disease is indicated by asterisks: $P < 0.05$ (*), and false discovery rate (FDR) < 0.05 (**). Scorecard information is provided after each protein name. The proteins referenced in the article are indicated with arrows.

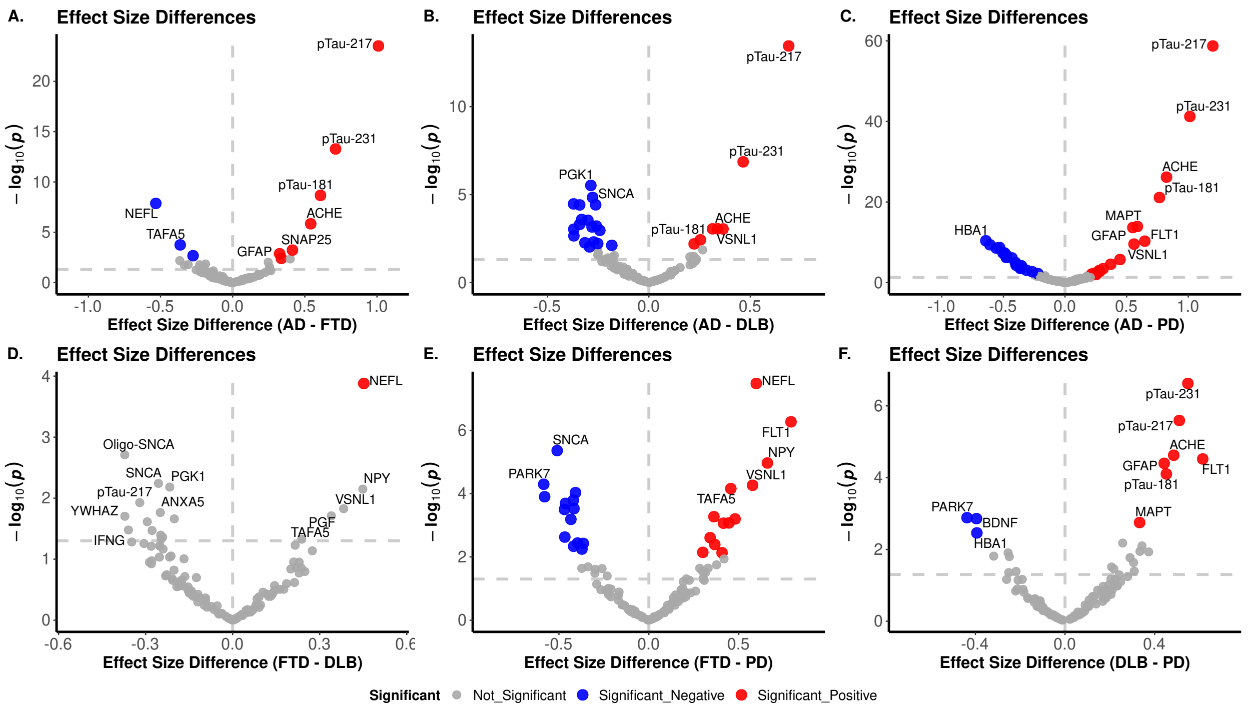

Supplementary Figure 4. Volcano Plots of Effect Size Differences across Diseases. (A-D) Volcano plot illustrating protein effect size differences between two neurodegenerative disease groups (e.g., AD vs. FTD) for each protein. The x-axis shows the difference in effect sizes between the groups, and the y-axis displays the -log10 transformed p-values from a z-test evaluating the significance of this difference, with p-values adjusted using the Benjamini–Hochberg method (FDR). Proteins with a significant positive difference (higher in the first group) are highlighted in red, those with a significant negative difference (higher in the second group) in blue, and non-significant proteins in gray.

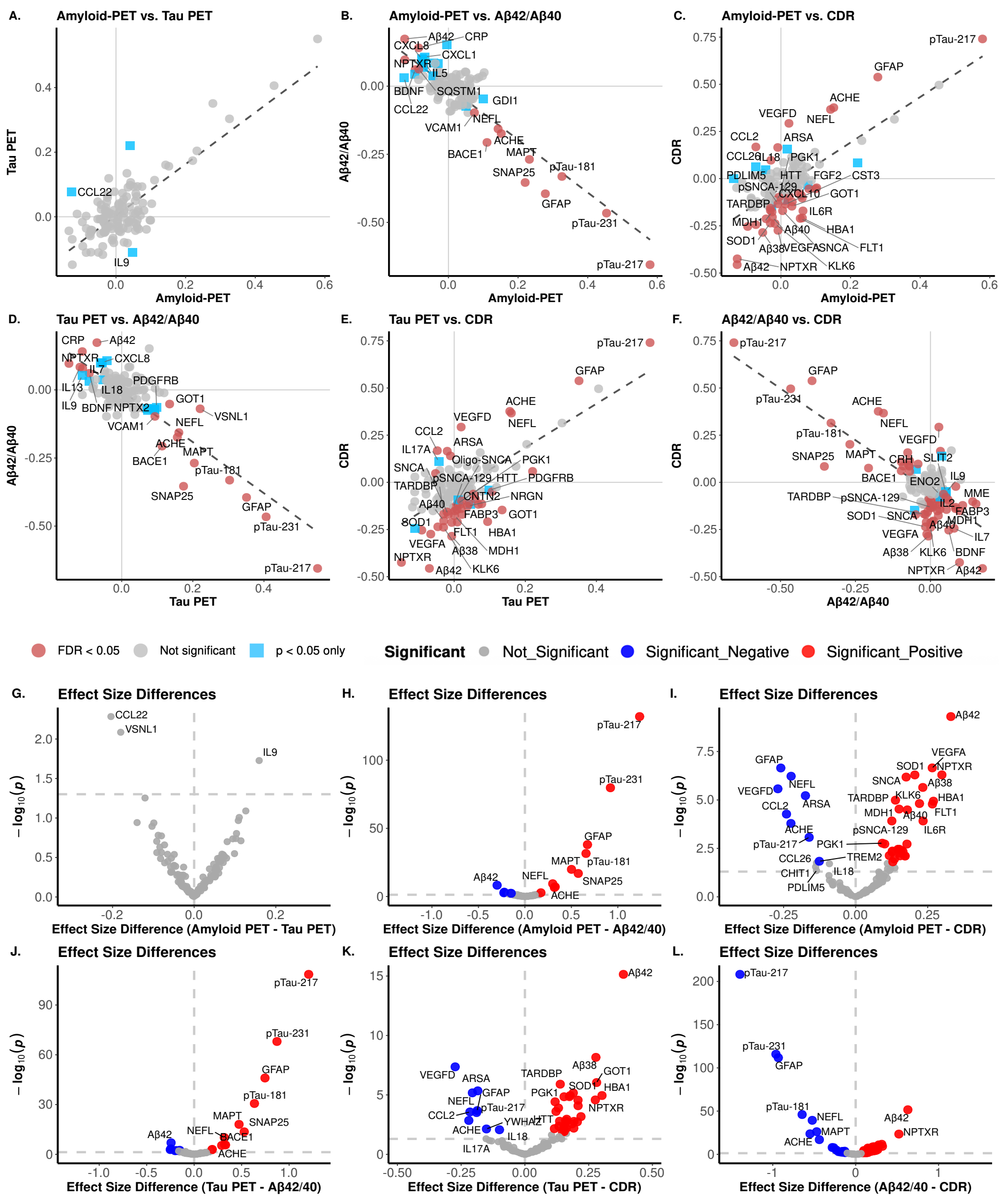

Supplementary Figure 5. Effect Size Analysis Across AD Phenotypes (A-F) Scatter plots comparing the effect sizes of 123 proteins across different AD phenotypes. Red circles indicate proteins that pass the FDR threshold (adjusted p-value < 0.05) from the z-test, with text labels added for the top 20 proteins with the smallest FDR values. Blue squares represent nominally significant proteins (p-value < 0.05 but not FDR-significant), with the top 5 (by smallest p-value) labeled. Grey circles indicate proteins that are not statistically significant. **(G-L)** These plots visualize results from z-tests comparing protein effect size estimates between two AD phenotypes (e.g., Amyloid PET vs. Tau PET). The x-axis shows the difference in effect sizes between the groups, and the y-axis displays the -log10 transformed p-values. Proteins significantly different between groups (FDR-adjusted p-value < 0.05) are highlighted in red (higher in first group) or blue (higher in second group). Non-significant proteins are shown in grey.

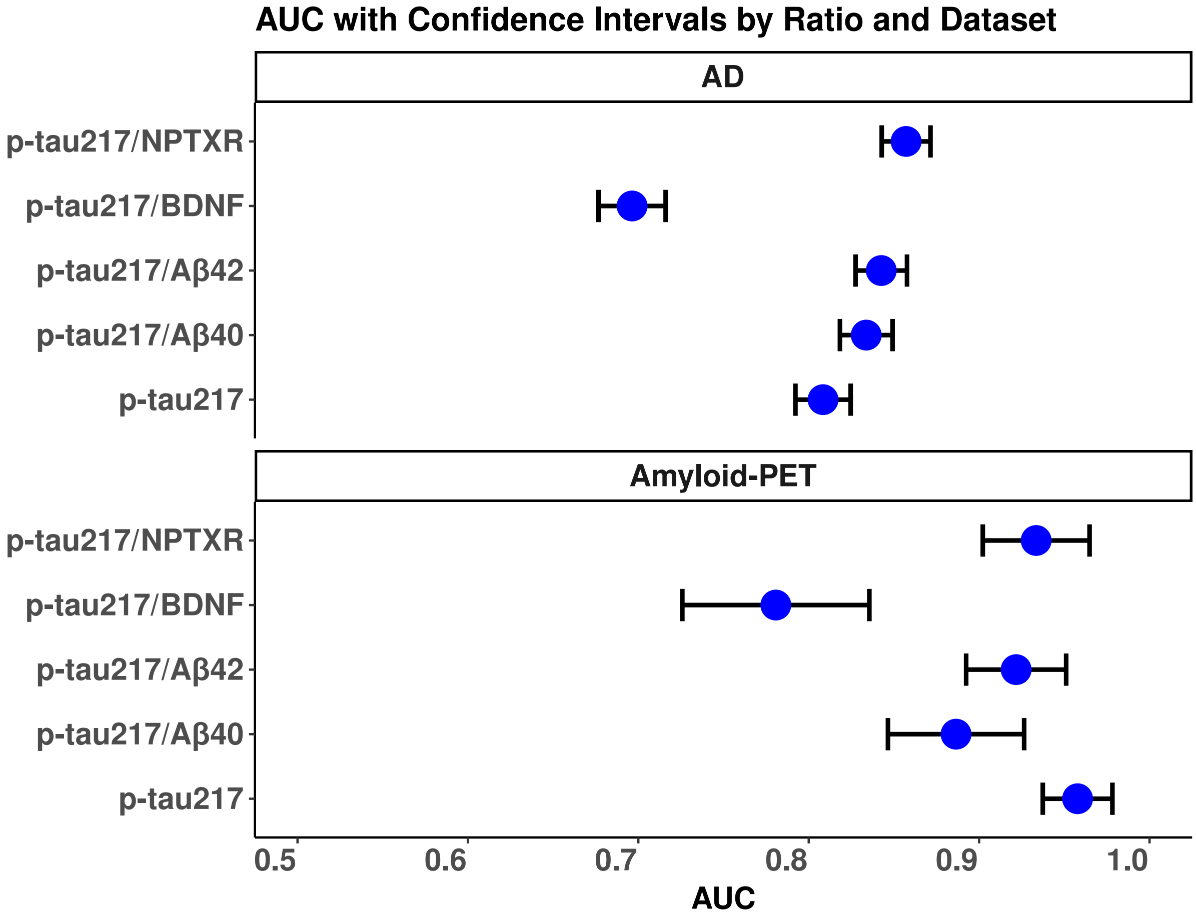

Supplementary Figure 6. Biomarker Ratios for AD Prediction and Amyloid-PET status. To evaluate the predictive potential of novel biomarkers, we analyzed p-tau217, p-tau217/Aβ40, p-tau217/Aβ42, p-tau217/BDNF, and p-tau217/NPTXR ratios to assess their ability to distinguish between Alzheimer's disease (AD) and cognitively unimpaired controls (CO), as well as to predict Amyloid-PET. The whisker plot compares the Area Under the Curve (AUC) values along with their corresponding 95% confidence intervals (CI) for each biomarker.

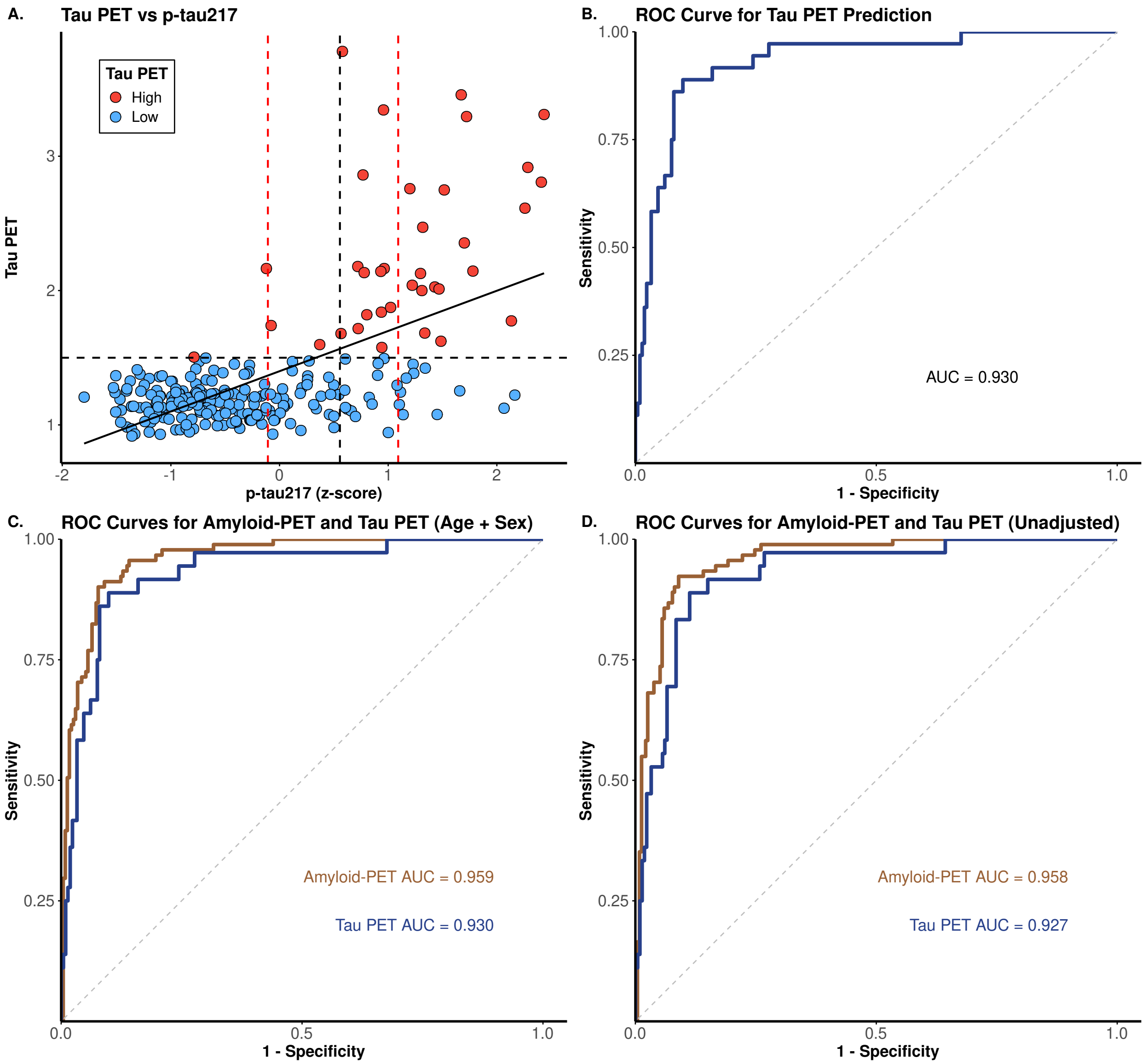

Supplementary Figure 7. p-tau217 as a Predictor of Tau PET and Amyloid-PET. (A) The scatterplot shows a positive correlation between p-tau217 and Tau PET, with Spearman's *rho* = 0.39 and $p = 2.70\times10^{-10}$. The vertical black dashed line marks Youden's Index single cutoff for plasma p-tau217. The intermediate range of plasma p-tau217 is highlighted by the lower and upper vertical red dashed lines, representing the thresholds corresponding to 95% sensitivity (lower line) and 95% specificity (upper line) for differentiating high from low Tau PET levels. **(B)** The ROC curve demonstrates the performance of p-tau217 in differentiating between high and low Tau PET levels (Threshold = 1.5), adjusted for age and sex. The blue line represents the ROC curve, with the dashed gray line indicating random chance. The Area Under the Curve (AUC) is 0.93, with a 95% confidence interval ranging from 0.886 to 0.974. **(C - D)** Comparison of the ROC curve for p-tau217 in distinguishing between Tau PET levels and Amyloid-PET (Centiloid Threshold = 20), with and without adjustment for age and sex.

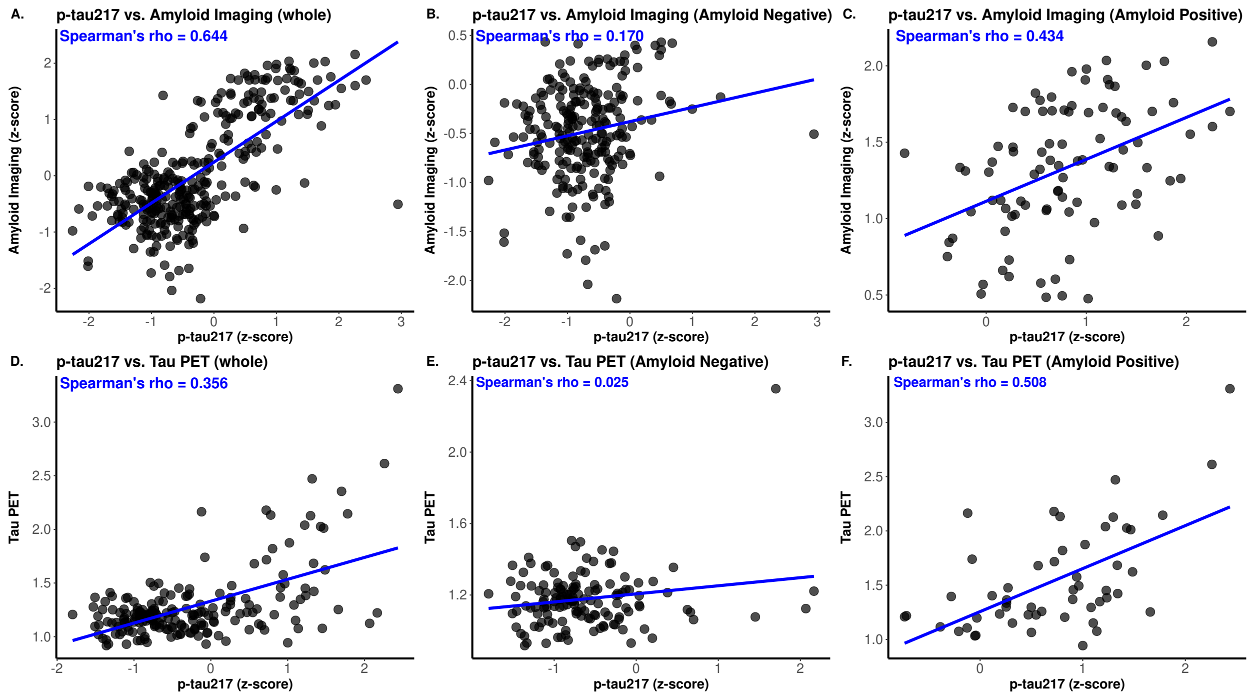

Supplementary Figure 8. Correlation of p-tau217 with Amyloid Imaging and Tau PET Across Distinct Groups. (A-C) The scatter plots illustrate the correlation between NULISAseq p-tau217 levels (x-axis) and Amyloid Imaging across three distinct groups, with both variables standardized using their log10 z-scores. The three groups include: (1) all samples, (2) amyloid imaging-negative samples, and (3) amyloid imaging-positive samples. A Spearman correlation line is overlaid in each plot to highlight the strength and direction of the non-parametric relationship between p-tau217 and amyloid imaging in each group. **(D-F)** A similar scatter plot is generated to explore the correlation between Tau PET imaging and p-tau217 levels across the same three groups, with the x-axis representing the z-score of log10-transformed p-tau217 values, while tauopathy was analyzed using the raw values. The Spearman correlation line is included in each graph to illustrate the association between p-tau217 and Tau PET.

A. Molecular Function

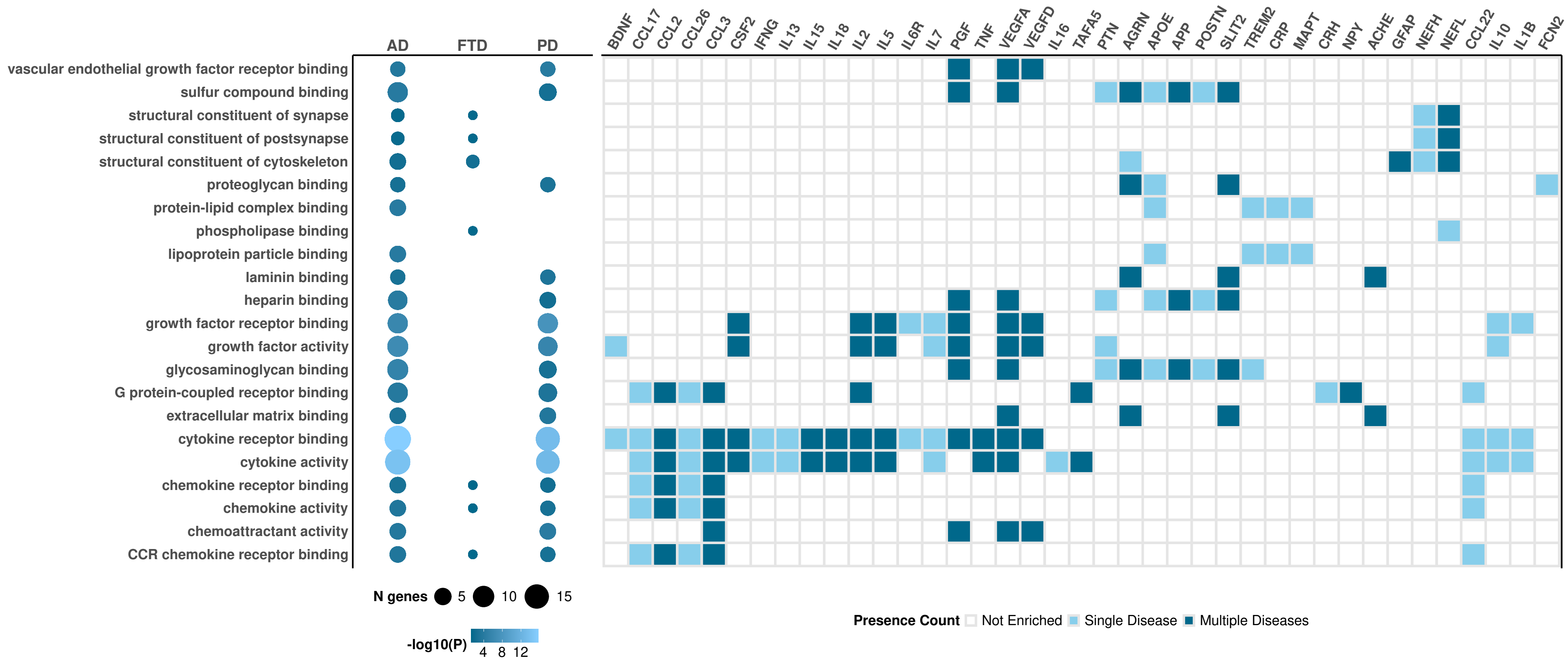

B. Cellular Component

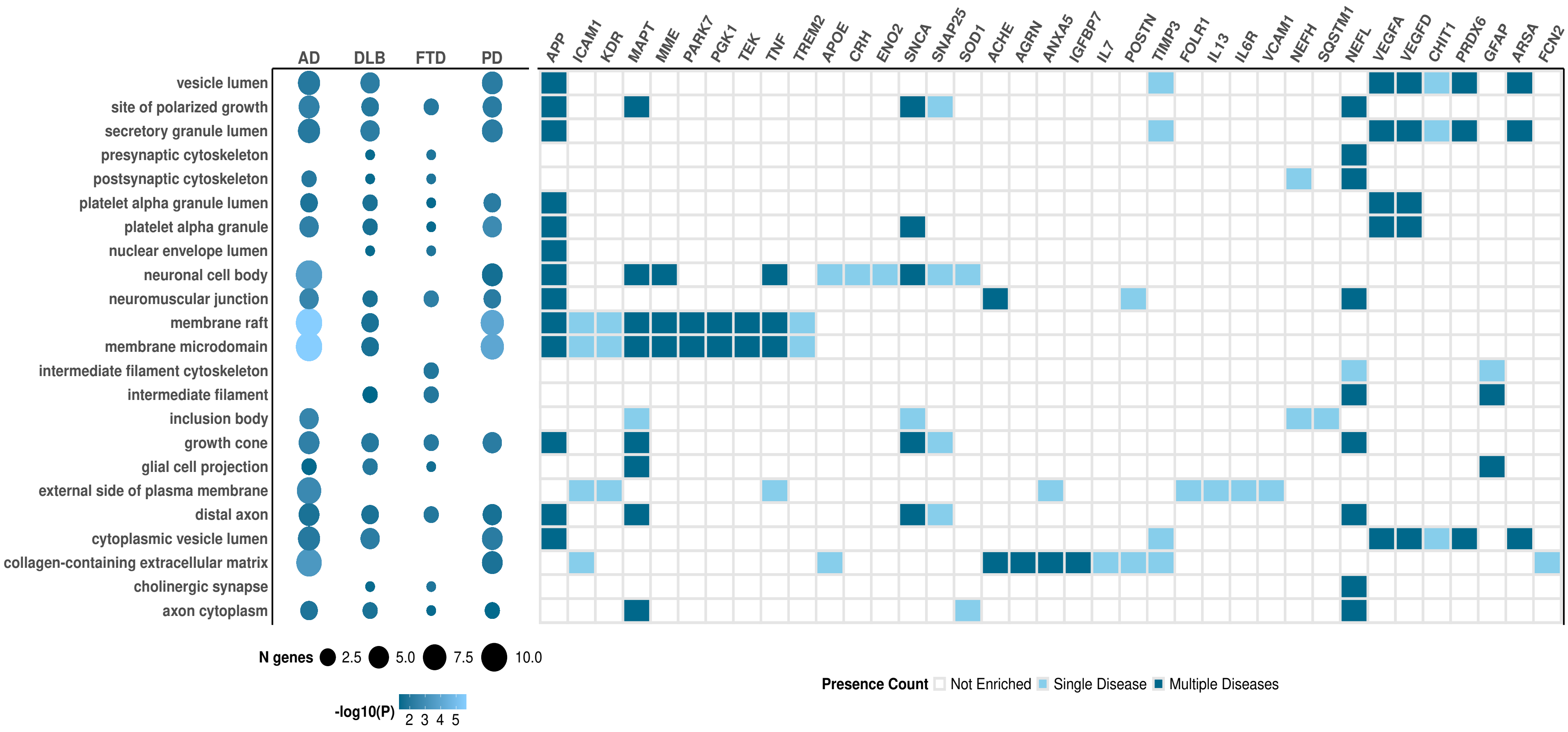

C. Biological Process

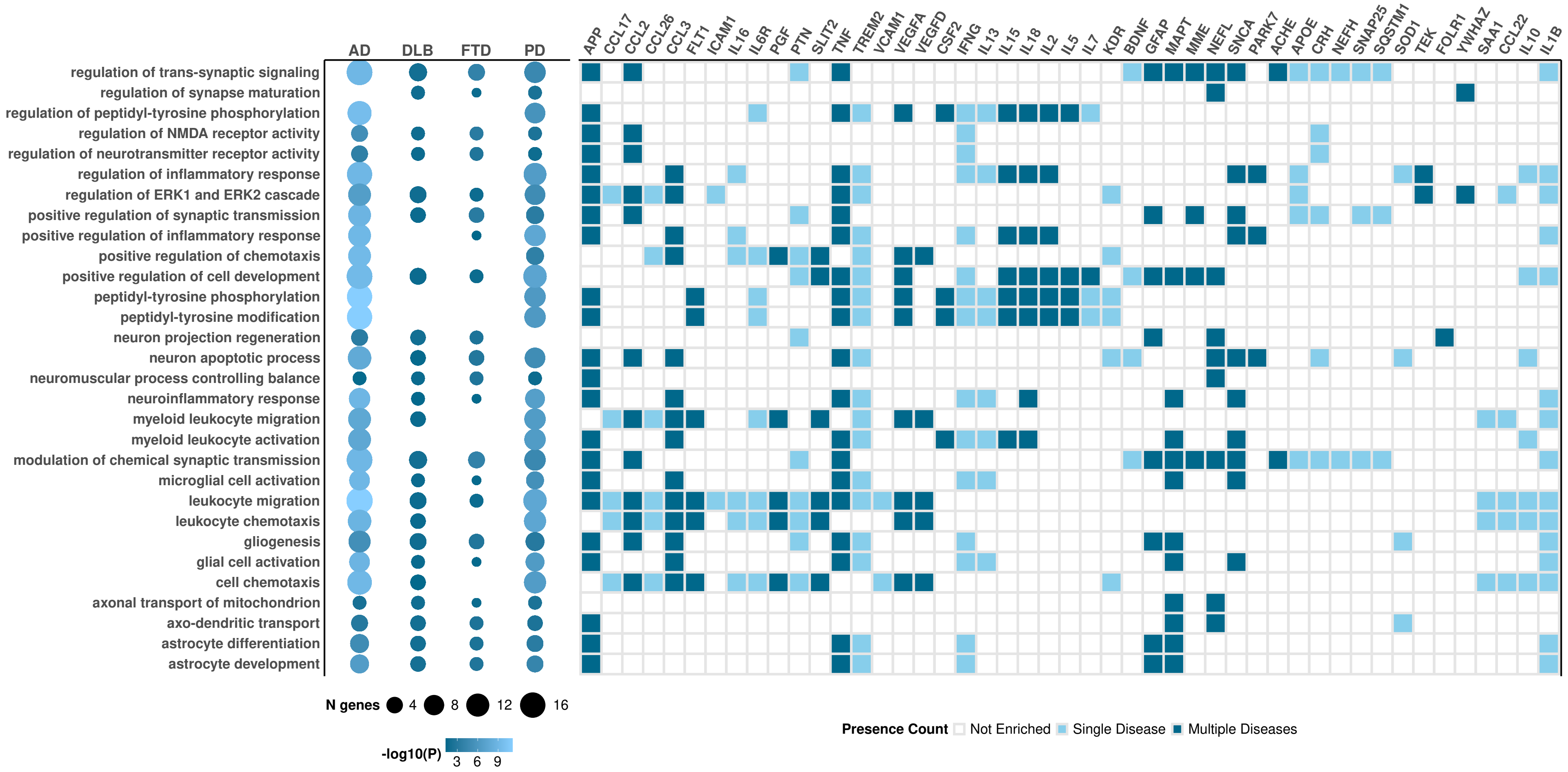

Supplementary Figure 9. Pathway Analysis. (A-C) Dot and Tile plot illustrating shared pathways and across patients expressed genes (DEGs) within each pathway with AD, DLB, FTD, and PD, categorized by Molecular Function, Cellular Component, and Biological Process. The pathways displayed represent the union of the top 10 most significant pathways for each disease, ordered by false discovery rate (FDR). The size of each dot corresponds to the number of genes identified in the respective pathway, while the color gradient reflects the level of FDR-corrected significance, with lighter colors indicating stronger statistical significance. The genes on the right are shaded based on pathway enrichment: dark blue indicates enrichment in multiple diseases, while light blue indicates enrichment in a single disease.

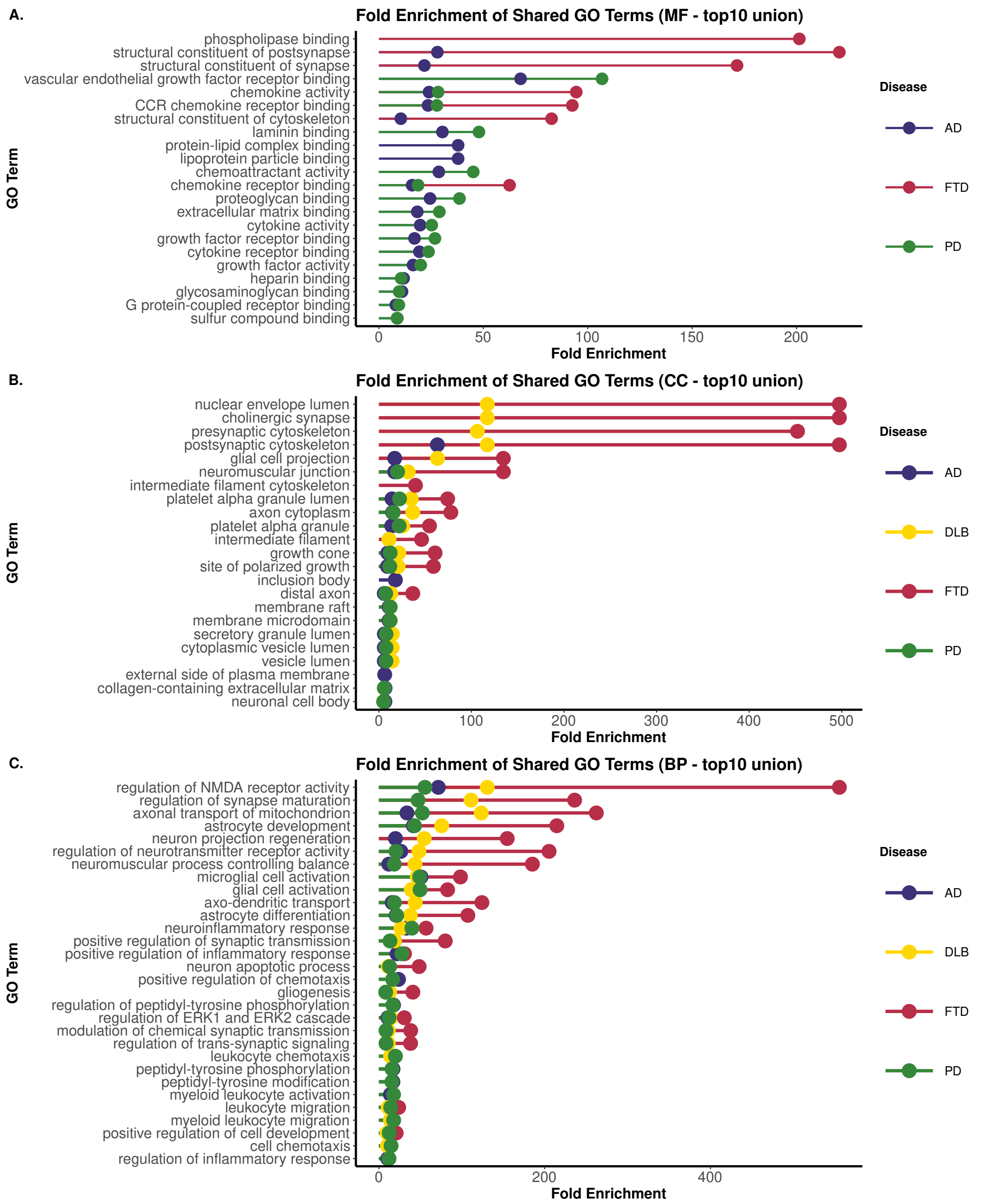

Supplementary Figure 10. Fold Enrichment of Shared GO Terms Across Diseases. This set of plots presents the fold enrichment of shared Gene Ontology (GO) terms across four disease groups (AD, DLB, FTD, and PD), analyzed separately for the Molecular Function (MF), Biological Process (BP), and Cellular Component (CC) categories. For each category, the top 10 GO terms (based on adjusted p-values) were selected separately for each disease, and the union of these terms across all diseases was used for visualization. Each line represents a GO term, with colored points indicating the fold enrichment for each disease. Colors represent diseases as follows: AD (dark purple), DLB (gold), FTD (deep red), and PD (green). Fold enrichment is calculated as the observed gene count relative to the background expectation.

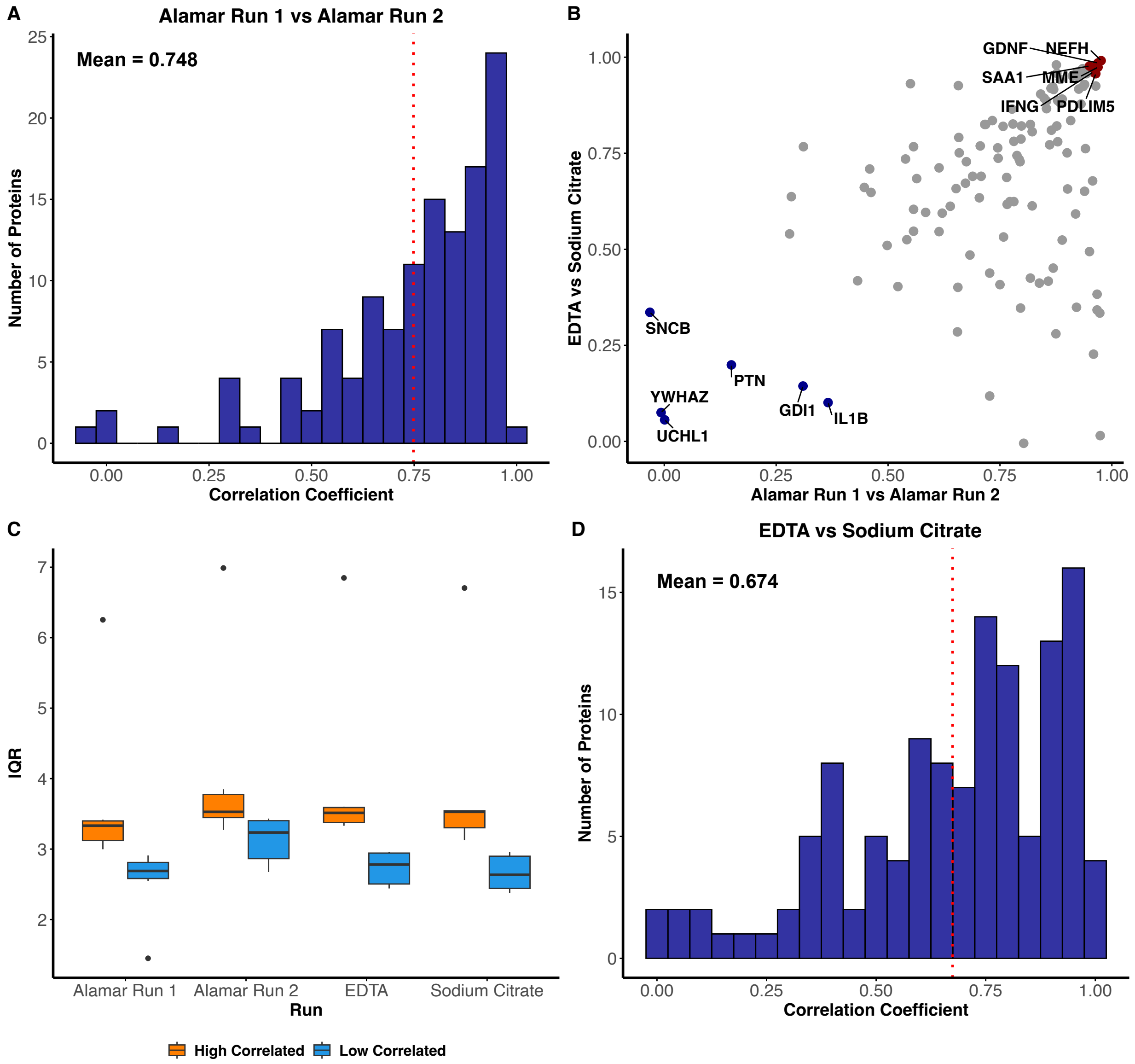

Supplementary Figure 11. Correlation between multiple Alamar runs. (A) The correlation distribution between Alamar Run 1 and Alamar Run 2 **(B)** A comparison of correlations for each protein between EDTA vs. Sodium Citrate and Alamar Run 1 vs. Alamar Run 2 **(C)** The relationship between correlation (*r*) and interquartile range (IQR) across the four runs. **(D)** The correlation distribution between EDTA and Sodium Citrate

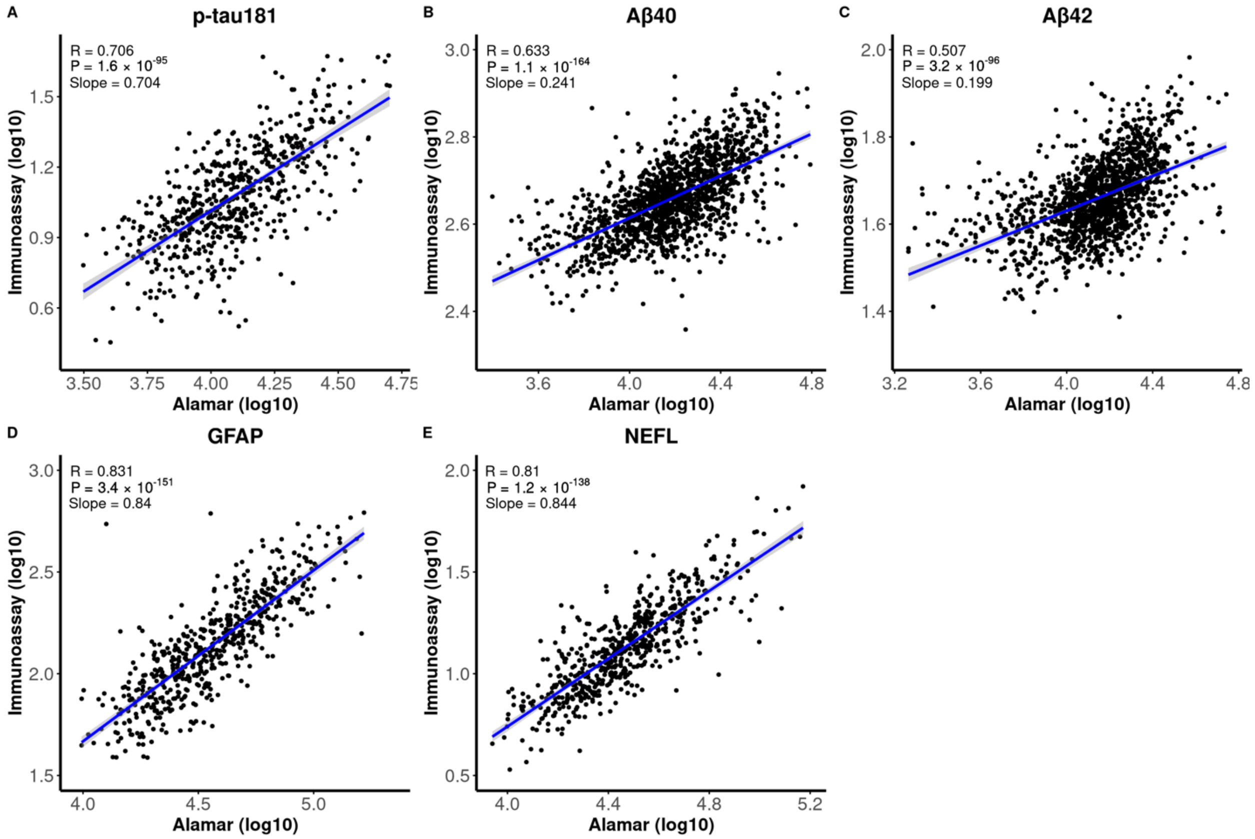

Supplementary Figure 12. Comparison of NULISAseq and Immunoassay Measurements. To validate the NULISA assay, we compared it against immunoassay-based proteomic measurements (*n* = 1,466) Immunoassay measurement was available for key proteins, including p-tau181, Aβ40, Aβ42, GFAP, NEFL. Scatter plots comparing plasma protein measurements obtained by NULISAseq™ and established immunoassay techniques across these overlapping samples. (A) p-tau181, (B) Aβ42, (C) Aβ40, (D) GFAP, and (E) NEFL. Spearman correlation lines are shown to indicate the strength and direction of associations between platforms.

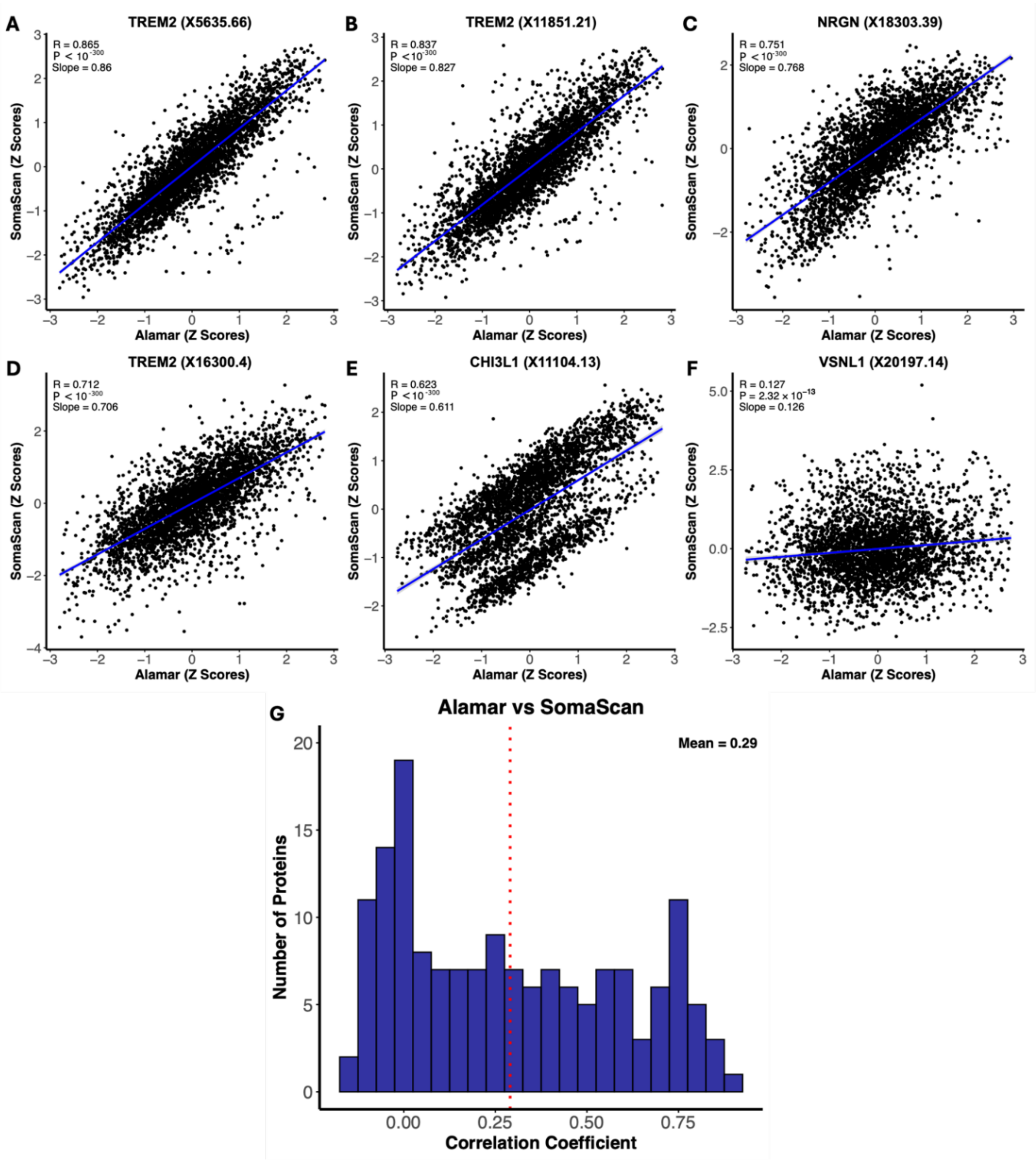

Supplementary Figure 13. Comparison between NULISAseq and SomaScan. Of the 123 CNS-related proteins measured by NULISAseq™, 112 have corresponding targets in the SomaLogic 7K panel, resulting in 148 analyte pairs due to multiple SomaScan assays for some proteins. **(A-F)** Scatter plots show scatter plots comparing plasma protein abundance measured by NULISAseq™ (Alamar Biosciences) and SomaScan across these overlapping targets in 3,716 samples. Each plot includes a Spearman correlation line to visualize the strength and direction of the relationship. The biomarkers analyzed are **(A)** TREM2 (X5635.66), **(B)** TREM2 (X11851.21), **(C)** Neurogranin (X18303.39), **(D)** TREM2 (X16300.4), **(E)** CHI3L1(X11104.13), and **(F)** VSNL1(X20197.14). These scatter plots provide insights into the concordance of measurements across platforms for these plasma biomarkers. **(G)** Correlation distribution between NULISAseq™ and SomaScan, summarizing overall concordance across platforms.
